# A Janus bacterial cellulose patch with antibacterial, antioxidant, anti-inflammatory and angiogenic activities for enhanced oral ulcer healing

**DOI:** 10.1093/rb/rbag160

**Published:** 2026-07-25

**Authors:** Guning Wang, Jingyu Yan, Chenying Cui, Lihong Zhou, Xiuping Wu, Bing Li

**Affiliations:** Shanxi Medical University School and Hospital of Stomatology and Shanxi Provincial Engineering Research Center for Oral Biomaterials, Taiyuan 030001, Shanxi, China; Shanxi Medical University School and Hospital of Stomatology and Shanxi Provincial Engineering Research Center for Oral Biomaterials, Taiyuan 030001, Shanxi, China; Shanxi Medical University School and Hospital of Stomatology and Shanxi Provincial Engineering Research Center for Oral Biomaterials, Taiyuan 030001, Shanxi, China; Shanxi Medical University School and Hospital of Stomatology and Shanxi Provincial Engineering Research Center for Oral Biomaterials, Taiyuan 030001, Shanxi, China; Shanxi Medical University School and Hospital of Stomatology and Shanxi Provincial Engineering Research Center for Oral Biomaterials, Taiyuan 030001, Shanxi, China; Shanxi Medical University School and Hospital of Stomatology and Shanxi Provincial Engineering Research Center for Oral Biomaterials, Taiyuan 030001, Shanxi, China

**Keywords:** Janus bacterial cellulose, oral ulcer, multifunctional patch, antibacterial, anti-inflammatory

## Abstract

Oral ulcers are complex mucosal lesions characterized by infection, inflammatory imbalance, elevated oxidative stress and impaired vascular regeneration, with therapeutic efficacy hindered by rapid drug loss in the highly wet and dynamic oral environment. Here, we developed a Janus-structured bacterial cellulose (BC)-based multifunctional oral patch (Ber@ZIF/BC, BZB) for stable retention and controlled release of Ber@ZIF (BZ) at ulcer sites. Encapsulation of berberine within ZIF and incorporation into Janus BC endowed the system with robust wet adhesion and microenvironment-responsive drug release. *In vitro*, BZB effectively inhibited *Staphylococcus aureus* and *Escherichia coli*, scavenged intracellular reactive oxygen species, preserved cell viability under oxidative stress and promoted epithelial and endothelial cell migration and angiogenesis while modulating inflammatory cytokine expression. In rat oral ulcer models, BZB accelerated ulcer closure, enhanced collagen remodeling and neovascularization and restored the inflammatory microenvironment. This Janus BZB patch synergistically regulates infection, oxidative stress and tissue regeneration, offering a promising strategy for precise, durable and efficient treatment of oral ulcers with translational potential.

## Graphical abstract

**Graphical Abstract:**
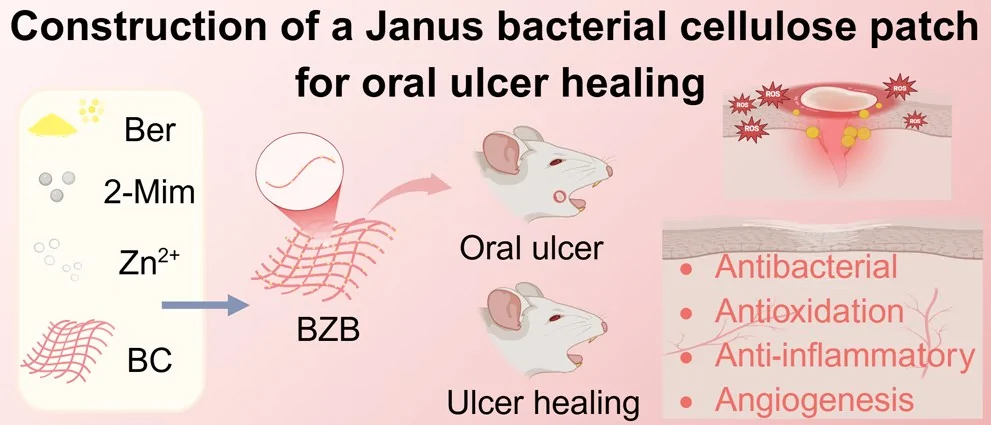

## Introduction

Oral ulcers comprise a heterogeneous group of mucosal lesions with diverse etiologies, including traumatic injury, chemical irritation, infection and immune-related disorders such as recurrent aphthous stomatitis. They are characterized by disruption of epithelial integrity, severe pain and a recurrent clinical course, which collectively impair mastication, speech and overall quality of life [1]. Although the precise pathogenesis has not been fully elucidated, accumulating evidence suggests that bacterial infection, dysregulated inflammatory responses, elevated oxidative stress and impaired angiogenesis act in concert to drive persistent epithelial damage and delayed mucosal healing [2].

At the pathological level, the development of oral ulcers is accompanied by disruption of the epithelial barrier, infiltration of inflammatory cells, excessive accumulation of reactive oxygen species (ROS) and enhanced microbial colonization [3, 4]. These alterations not only aggravate local tissue damage but also establish a detrimental microenvironment that impedes re-epithelialization and tissue remodeling [5]. Current clinical management primarily relies on the topical administration of corticosteroids, antibiotics, anesthetics or immunomodulatory agents to alleviate inflammation and pain [6–8]. However, such single-target therapeutic strategies are often insufficient to simultaneously address the multifactorial nature of the disease, including infection, inflammatory dysregulation and oxidative stress [9]. Moreover, prolonged use may lead to adverse effects such as mucosal atrophy, microbial dysbiosis and the development of drug resistance.

Local drug delivery is widely regarded as the most effective therapeutic route for oral ulcers; however, its efficacy is largely constrained by the unique oral microenvironment. Unlike cutaneous wounds, the oral mucosa is continuously exposed to a highly hydrated and dynamic setting, where persistent saliva secretion, swallowing, mastication and speech movements rapidly dilute or remove exogenous agents [10]. As a result, conventional formulations such as mouthwashes, sprays, gels and standard patches typically lose effectiveness within tens of minutes to a few hours [11]. In addition, the fibrinous pseudomembrane covering the ulcer surface acts as a physical barrier that limits drug penetration into deeper lesion sites, further reducing therapeutic efficiency [12]. Therefore, an ideal therapeutic material for oral ulcers should not only exhibit robust wet adhesion and prolonged retention at the lesion site, but also provide sustained antibacterial and anti-inflammatory effects, mitigate oxidative stress and promote angiogenesis, thereby establishing a favorable microenvironment for mucosal regeneration [13].

Recent advances in oral ulcer management have led to the development of various local therapeutic platforms, including hydrogels, electrospun nanofibrous membranes, mucoadhesive films and multifunctional oral patches. Hydrogels possess favorable biocompatibility and drug loading capacity; however, their highly hydrated nature often results in excessive swelling, weak mechanical stability and limited retention under continuous salivary flushing [14–17]. Electrospun membranes and thin films can mimic extracellular matrix-like architectures and support drug incorporation, yet they commonly suffer from insufficient wet adhesion and rapid drug diffusion in the dynamic oral environment [18–20]. Mucoadhesive patches have been developed to prolong local retention through interactions with mucins or salivary glycoproteins, but most existing systems primarily focus on adhesion enhancement while lacking the ability to simultaneously regulate bacterial infection, inflammation, oxidative stress and tissue regeneration [21, 22]. More recently, Janus-structured materials with asymmetric functional interfaces have attracted increasing attention because they enable spatial separation of adhesive and therapeutic functions within a single platform [23]. Nevertheless, many previously reported oral ulcer patches or Janus systems still fail to simultaneously achieve durable wet adhesion, salivary erosion resistance, stimuli-responsive release and multifunctional therapeutic regulation within a single platform. Therefore, the development of a biomimetic oral patch that combines stable wet adhesion, resistance to salivary erosion, sustained local drug release and multifunctional microenvironment modulation remains highly desirable.

Among a wide range of naturally derived bioactive molecules, berberine (Ber) is an isoquinoline alkaloid extracted from various medicinal herbs that has been extensively utilized for the treatment of infection and inflammation. Modern pharmacological studies have further demonstrated that berberine exhibits broad-spectrum antibacterial, anti-inflammatory, antioxidant and immunomodulatory activities [24]. These effects are primarily mediated through disruption of bacterial cell membranes, interference with protein and DNA synthesis and regulation of inflammation-related signaling pathways, thereby effectively alleviating infection-associated and inflammation-driven tissue damage [25]. In addition, berberine contributes to the restoration of mucosal barrier function by maintaining epithelial tight junction integrity, inhibiting pathogen adhesion and modulating local microbial homeostasis [26]. These properties make it a promising therapeutic candidate for oral ulcers, which are characterized by the coexistence of infection, inflammation and barrier disruption [27].

However, the clinical translation of berberine in oral applications is significantly hindered by its poor aqueous solubility and limited local bioavailability [28]. In the highly hydrated oral environment, free berberine is readily dispersed and cleared by saliva, making it difficult to maintain an effective therapeutic concentration at the ulcer site, thereby markedly compromising its therapeutic efficacy [29]. Therefore, the development of a delivery system capable of stably encapsulating berberine and enabling controlled release is essential to fully exploit its multifaceted pharmacological potential.

Metal-organic frameworks (MOFs) have emerged as promising platforms for the delivery of poorly soluble therapeutics due to their high specific surface area, tunable pore structure and superior drug loading capacity [30, 31]. Among them, zeolitic imidazolate framework-8 (ZIF-8, hereafter referred to as ZIF), formed through the coordination of Zn^2+^ ions with 2-methylimidazole, exhibits favorable structural stability and acceptable biocompatibility within appropriate dosage ranges, together with pH-responsive degradation behavior under acidic inflammatory conditions [32–34]. Encapsulation of berberine within the ZIF nanostructure to form Ber@ZIF, abbreviated as BZ, significantly improves its dispersion and stability in aqueous environments. More importantly, in the mildly acidic microenvironment of oral ulcers, the framework undergoes controlled disassembly, triggering the release of berberine. This process is accompanied by the liberation of Zn^2+^ ions, which possess intrinsic antibacterial activity and can act synergistically with berberine. As a result, the system enables coordinated antibacterial, anti-inflammatory and antioxidative effects, thereby contributing to the regulation of the pathological microenvironment.

Although BZ effectively improves the solubility and stimuli-responsive release of berberine, its therapeutic performance in the oral cavity remains highly dependent on the retention capability of the carrier on the mucosal surface and its resistance to salivary clearance [35]. The healthy oral mucosa possesses a multilayered barrier architecture, in which the surface mucus layer provides both anti-fouling and lubricating functions, thereby preventing bacterial adhesion and buffering continuous mechanical stimulation. Within this highly hydrated and dynamically changing environment, stable adhesion and interfacial integrity are critical for maintaining mucosal homeostasis and promoting tissue repair [36].

Inspired by the structural features of native biological interfaces, Janus-structured biomaterials with asymmetric physicochemical properties have recently attracted considerable attention in diverse tissue repair applications, including skin wound healing, osteochondral regeneration, vascular repair and localized drug delivery [37]. Owing to their spatially separated functional interfaces, Janus systems can integrate distinct or even opposing properties within a single platform, such as wet adhesion versus barrier protection or sustained drug release versus mechanical stability, thereby enabling more precise regulation of complex pathological microenvironments [38]. Particularly in highly hydrated and dynamic biological settings, Janus architectures have demonstrated significant advantages in improving interfacial stability, directional therapeutic delivery and tissue regeneration efficiency [39]. These characteristics make Janus-structured materials especially suitable for oral ulcer therapy, where prolonged mucosal adhesion, resistance to salivary erosion and sustained local treatment are simultaneously required [40]. However, despite these advances, the application of Janus-structured biomaterials in oral ulcer therapy remains relatively underexplored, particularly for systems capable of integrating durable wet adhesion with stimuli-responsive multifunctional therapy. Notably, bacterial cellulose (BC) inherently possesses an asymmetric dense and porous architecture, which closely matches the design requirements of Janus systems. The dense surface layer can provide stable wet adhesion and protective barrier functions, whereas the porous layer facilitates drug loading and sustained release. Such intrinsic structural asymmetry makes BC an attractive natural platform for constructing biomimetic oral Janus patches with spatially differentiated functions through relatively simple fabrication processes [41]. To further address the limitations of mucosal retention and local drug delivery, BZ was incorporated into a Janus BC matrix. The dense BC layer, composed of an entangled nanofibrous network, provides stable wet adhesion and resistance to salivary erosion, whereas the porous layer facilitates efficient BZ loading and sustained release. Such asymmetric structural integration enables simultaneous enhancement of mucosal retention and localized therapeutic delivery in the dynamic oral environment.

In this study, we developed a biomimetic Janus-structured BC-based oral patch to address the long-standing challenges of insufficient mucosal retention and ineffective multifunctional therapy in oral ulcer treatment. By integrating Ber@ZIF (BZ) into the porous layer of BC while preserving the dense adhesive interface, a spatially compartmentalized therapeutic platform (BZB) was successfully constructed. Unlike previously reported homogeneous oral patches or single functional adhesive systems, the present Janus platform simultaneously integrates stable wet adhesion, salivary erosion resistance, pH-responsive release and multifunctional microenvironment regulation within a single biomimetic construct. In addition, the intrinsic asymmetric structure of BC provided a simple yet effective strategy for fabricating multifunctional Janus patches without complicated interfacial engineering processes. Upon exposure to the acidic inflammatory microenvironment of oral ulcers, the BZ nanostructure underwent controlled degradation, triggering the sustained release of berberine and Zn^2+^, thereby enabling synergistic regulation of infection, inflammation, oxidative stress and tissue repair within the ulcer microenvironment (Figure 1). Collectively, this work presents a multifunctional biomimetic oral patch that integrates asymmetric interfacial design with stimuli-responsive therapeutic regulation, thereby offering a promising strategy for sustained local therapy and accelerated mucosal regeneration under highly dynamic oral conditions.

**Figure 1:**
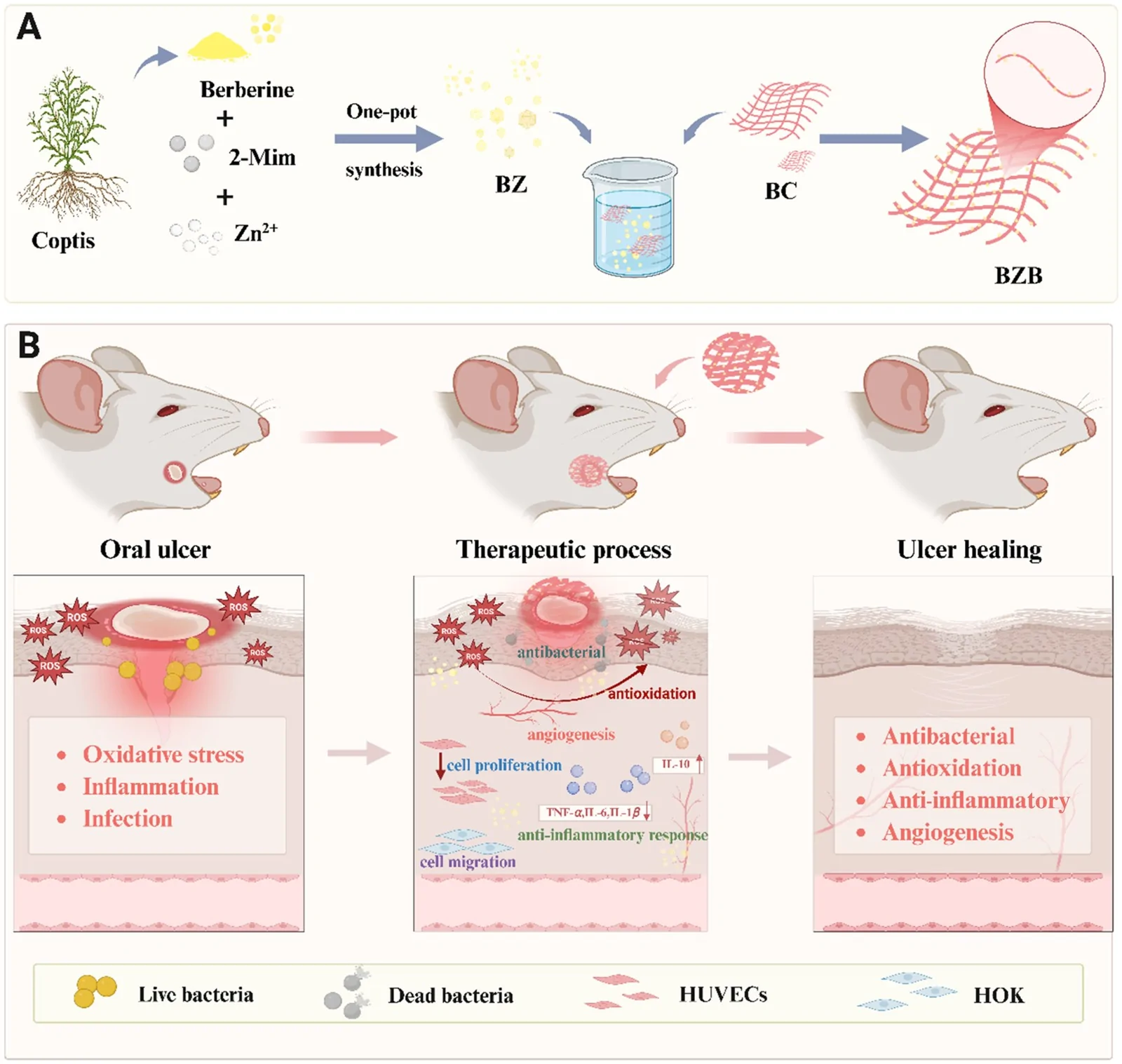
Schematic illustration of the fabrication of the Janus-structured BZB patch and its multifunctional therapeutic effects in oral ulcer healing. (A) Schematic illustration of the synthesis of berberine-loaded ZIF nanoparticles (BZ) and their incorporation into the Janus BC matrix to construct the BZB patch. (B) Schematic illustration of the therapeutic process of BZB in oral ulcer healing, highlighting its antibacterial, antioxidant, anti-inflammatory, and pro-angiogenic effects.

## Materials and methods

### Preparation of BZ nanoparticles

BZ nanoparticles were synthesized via a one-step *in situ* self-assembly method. Briefly, zinc acetate (≥99.0%, MW: 219.5, CAS: 5970-45-6) was dissolved in methanol (≥99.9%, MW: 32.04, CAS: 67-56-1) to obtain a 0.05-M solution, while 2-methylimidazole (≥99.0%, MW: 82.10, CAS: 693-98-1) was dissolved in methanol to form a 0.4-M solution, corresponding to a molar ratio of Zn^2+^ to ligand of 1:8. Subsequently, berberine (≥99.0%, MW: 336.36, CAS: 2086-83-1) at a concentration of 1 mg/mL was introduced into the 2-methylimidazole solution and magnetically stirred at room temperature for 10 min to obtain a homogeneous dispersion. The zinc precursor solution was then rapidly added to the ligand solution containing berberine, followed by continuous stirring at ∼800 rpm for 60 min at room temperature. During this process, Zn^2+^ coordinated with 2-methylimidazole to form the ZIF framework, while berberine was simultaneously encapsulated within the growing nanocrystals. After completion of the reaction, the resulting mixture was centrifuged at 10 000 rpm for 10 min to collect the precipitate. The product was washed three times with methanol and deionized water to remove unreacted precursors and surface-adsorbed free drug. Finally, the obtained nanoparticles were dried under vacuum for 12 h to yield BZ nanoparticles.

### Preparation of BC and BZB

BC was first produced by culturing *Komagataeibacter sp*. Briefly, the bacteria were incubated at 30°C and 200 rpm for 24 h, followed by inoculation into a static fermentation medium and incubation at 30°C to form BC membranes, which were harvested every 24 h. The obtained BC was thoroughly washed with deionized water, then boiled in 0.1 M NaOH solution to remove bacterial residues and impurities and repeatedly rinsed to neutral pH to obtain purified BC. To construct the Janus structure, one side of the purified BC membrane was exposed to the air interface to form a dense layer, while the opposite side remained in contact with the culture medium to form a porous layer, resulting in a bilayer BC substrate with asymmetric microstructure and surface properties.

Subsequently, BZ was incorporated as the functional component to prepare composite hydrogels. BC (3 g, dry weight) was dispersed in 30 mL ultrapure water, followed by the addition of 10 mL BZ dispersions at different concentrations of 5, 10 and 20 mg/mL, yielding corresponding BZ-loaded Janus BC systems denoted as BZB1, BZB2 and BZB3. These concentrations were selected based on preliminary optimization experiments to evaluate the concentration-dependent effects of BZ incorporation on the structural properties, biological activity and cytocompatibility of the composite system. The mixtures were incubated at 30°C with shaking at 160 rpm to facilitate nanoparticle infiltration into the porous layer. The incubation was continued for 24 h to achieve stable embedding of nanoparticles within the BC network and effective interfacial integration. After incubation, the composite hydrogels were thoroughly rinsed with deionized water to remove unbound particles, resulting in Janus-structured BZB composite hydrogels.

To meet the requirements for preclinical evaluation, the Janus BC and BZB hydrogel patches were sterilized before *in vitro* and *in vivo* experiments. Briefly, the prepared patches were carefully packaged in sterile sealed bags and subjected to ethylene oxide (EO) sterilization. The sterilization conditions were 55°C, 60% relative humidity and an exposure time of 6 h, followed by 48 h of aeration to remove residual EO.

### Morphological and structural characterization

Scanning electron microscopy (SEM) was employed to observe the microstructure and morphological features, with particular emphasis on particle size, distribution uniformity and the Janus asymmetric architecture of BC. Dynamic light scattering (DLS) was used to determine the hydrodynamic diameter and size distribution in deionized water. The surface charge properties were measured using a zeta potential analyzer. Ultraviolet-visible (UV-vis) spectroscopy was applied to assess the loading of berberine. ZIF and BZ were dispersed in deionized water and scanned over the wavelength range of 200–500 nm to identify the characteristic absorption peaks of berberine at 345 and 425 nm and their corresponding changes. Fourier-transform infrared (FT-IR) spectroscopy was conducted to analyze the chemical structure and functional groups, with a scanning range of 400–4000 cm^−1^. X-ray photoelectron spectroscopy (XPS) was utilized to investigate the elemental composition and chemical states on the sample surface. X-ray diffraction (XRD) analysis was performed to characterize the crystalline structure over a scanning range of 5°–40°.

To evaluate the structural persistence and oral environmental stability of the composite materials, BZB was immersed in either phosphate-buffered saline (PBS) or artificial saliva and subjected to different conditions, including static incubation for 24 h, magnetic stirring at 300 rpm and ultrasonic treatment at 100 W. The samples were then collected and weighed before and after drying, and the residual weight ratio (RWR) was calculated to assess their resistance to degradation and structural integrity. The interfacial wettability of the materials was characterized using a contact angle measurement system. At room temperature, a defined-volume of water droplet of ∼5 μL was placed on the surface of BC and BZB, and the dynamic changes in contact angle were recorded from 0 to 60 s to analyze surface hydrophilicity and water penetration behavior.

To determine the drug loading content (DLC) and encapsulation efficiency (EE) of BZ nanoparticles, the amount of non-encapsulated berberine in the supernatant was quantified using UV-vis spectroscopy at 345 nm. Briefly, after synthesis, the reaction mixture was centrifuged and the collected supernatants were combined. The concentration of free berberine was determined according to an established calibration curve. The encapsulation efficiency (EE) and drug loading content (DLC) were calculated as follows:


$$\begin{array}{c} EE(\%)=\frac{W_{0}-W_{f}}{W_{0}}\times 100\%\\ D\mathrm{LC}(\%)=\frac{W_{0}-W_{f}}{W_{\mathrm{BZ}}}\times 100\% \end{array},$$

where *W*_0_ represents the initial amount of berberine added during synthesis, *W*_f_ represents the amount of free berberine detected in the supernatant, and *W*_BZ_ represents the total mass of collected BZ nanoparticles.

To quantify the actual berberine content in BZB patches, ∼50 mg of freeze-dried BZB was immersed in 10 mL of 0.1 M HCl and stirred for 12 h to ensure complete decomposition of the ZIF framework and quantitative release of encapsulated berberine. After centrifugation, the absorbance of the supernatant was measured at 345 nm using UV-vis spectroscopy. The berberine concentration was calculated according to the standard calibration curve, and the berberine content was expressed as milligrams of berberine per gram of dry patch.

To simulate the salivary flushing and mechanical stimulation conditions of the oral cavity, BC and BZB patches were subjected to artificial saliva soaking, stirring and sonication treatments before being applied to the surface of freshly excised porcine tongue mucosa for adhesion testing. After allowing sufficient contact between the samples and the tissue, the maximum detachment force during debonding was measured using a universal testing machine. This value was used to evaluate the wet adhesion performance and interfacial stability of the patches under dynamic oral conditions.

### *In vitro* release behavior

The release profiles of berberine from BZ and BZB were investigated in PBS (pH 7.4) at 37°C under continuous shaking. Briefly, a predetermined amount of sample was immersed in release medium and incubated under sink conditions. At designated time points, aliquots of the supernatant were collected for analysis and immediately replenished with an equal volume of fresh PBS. The concentration of released berberine was determined by UV-vis spectroscopy at 345 nm using the previously established calibration curve. The cumulative release percentage was calculated based on the total berberine content of each formulation. All measurements were performed in triplicate.

### Free radical scavenging assay

The antioxidant activity of BZ was evaluated using ABTS•^+^ and DPPH• radical scavenging assays. The ABTS•^+^ radical cation solution was prepared by reacting ABTS with an oxidizing agent and allowing the mixture to stand in the dark until stabilization. During the assay, BZ at concentrations of 3 and 6 mg/mL was added to the ABTS•^+^ radical solution, and the change in absorbance was measured at 734 nm. For the DPPH• assay, BZ at concentrations of 1 and 3 mg/mL was mixed with a DPPH solution in ethanol and incubated in the dark for 30 min, after which the absorbance was measured at 517 nm. The free radical scavenging efficiency was calculated based on the change in absorbance, thereby assessing the antioxidant capacity of the material.

### *In vitro* antibacterial activity of BZB

The antibacterial activity of BZB was evaluated using *Staphylococcus aureus* (*S.aureus*) and *Escherichia coli* (*E.coli*) as representative Gram-positive and Gram-negative bacterial strains, respectively. Both strains were cultured in Luria–Bertani (LB) medium broth at 37°C with shaking at 200 rpm to the logarithmic growth phase, and the bacterial concentration was adjusted to ∼1 × 10^6^ CFU/mL using spectrophotometric measurement. Equal volumes of bacterial suspension were added into 24-well plates, followed by the introduction of different material groups. The samples were incubated at 37°C under mild shaking conditions for 12 h to simulate the interaction between materials and bacteria in the oral microenvironment. After incubation, 100 μL of bacterial suspension from each group was collected, subjected to tenfold serial dilution and evenly spread onto LB medium agar plates. The plates were incubated at 37°C for 24 h, and colony-forming units were counted to quantitatively assess antibacterial efficacy. To further evaluate bacterial viability, a SYTO-9 and propidium iodide (PI) dual staining kit was employed for live/dead fluorescence staining. The samples were then observed under a fluorescence microscope to record the distribution of green fluorescence indicating live bacteria and red fluorescence indicating dead bacteria.

Bacterial morphological changes were examined using SEM. After treatment, bacterial samples were washed three times with PBS to remove unbound materials, followed by fixation with 2.5% glutaraldehyde for 2 h. The samples were then dehydrated through a graded ethanol series of 30%, 50%, 70%, 90% and 100%, with each step lasting 10 min. After critical point drying and sputter coating, the bacterial surface morphology and structural integrity were observed under SEM.

### Biocompatibility evaluation

The cytocompatibility of the materials was assessed using human oral keratinocytes (HOK) *in vitro*. A Calcein-AM and PI dual staining assay was performed to evaluate cell viability. The distribution of green fluorescence indicating live cells and red fluorescence indicating dead cells was observed under a fluorescence microscope, providing a qualitative assessment of cytotoxicity and cell growth status.

Hemocompatibility was evaluated using a hemolysis assay. Fresh anticoagulated whole blood was diluted with PBS to obtain a red blood cell suspension. Different materials were incubated with the red blood cell suspension at 37°C for 2 h. Deionized water-treated samples were used as the positive control representing complete hemolysis, while PBS-treated samples served as the negative control. After incubation, the samples were centrifuged at 3000 rpm for 5 min, and the supernatant was collected for absorbance measurement at 540 nm.

### Intracellular ROS detection and antioxidative protection assay

To simulate oxidative stress conditions in the oral ulcer microenvironment, H_2_O_2_ was used to induce intracellular ROS generation in human umbilical vein en dothelial cells (HUVECs). Except for the Blank group, all groups were treated with H_2_O_2_ at a concentration of 200 μM for 4 h to establish an oxidative stress model. Subsequently, the corresponding materials were added and incubation was continued for 12 h to evaluate their protective effects against oxidative damage. Intracellular ROS levels were detected using the DCFH-DA fluorescent probe, and the green fluorescence intensity was observed under a fluorescence microscope to qualitatively assess ROS variations among different groups.

Based on the H_2_O_2_-induced oxidative stress model, cell viability was further evaluated using a CCK-8 assay. After H_2_O_2_ stimulation for 4 h, the culture medium was replaced with fresh medium containing different materials, and cells were incubated until Day 1 and Day 3. The Blank group was used as the reference to calculate cell viability, thereby assessing the ability of the materials to alleviate oxidative damage and provide cytoprotective effects under oxidative stress conditions.

### Regulation of inflammatory microenvironment

To evaluate the regulatory effects of different materials on the inflammatory microenvironment, an *in vitro* inflammation model was established using lipopolysaccharide (LPS)-stimulated RAW264.7 cells. All experimental groups were treated with LPS at a concentration of 1 μg/mL for 12 h to induce an inflammatory response. The control group received LPS stimulation only without any material treatment, while the other groups were co-cultured with corresponding materials during LPS stimulation. After treatment, the culture medium was discarded and the cells were gently washed with PBS. Total RNA was extracted using TRIzol reagent, followed by reverse transcription to obtain cDNA according to the manufacturer instructions. Reverse transcription-quantitative polymerase chain reaction (RT-qPCR) was then performed to analyze the expression levels of inflammation-related genes, including pro-inflammatory cytokines TNF-*α*, IL-6 and IL-1*β*, as well as the anti-inflammatory cytokine IL-10. The primer sequences are listed in Supplementary Table S1. Gene expression levels were calculated using the 2^−ΔΔCt^ method and normalized to GAPDH as the internal reference gene.

To further verify the inflammatory regulatory effects at the protein level, immunofluorescence (IF) staining was performed to evaluate the expression of inducible nitric oxide synthase (iNOS) in RAW264.7 cells. After LPS stimulation and treatment with different materials, cells were fixed with 4% paraformaldehyde, permeabilized with 0.1% Triton X-100 and blocked with 5% bovine serum albumin. Subsequently, cells were incubated with primary antibody against iNOS at 4°C overnight, followed by incubation with a fluorescently labeled secondary antibody for 1 h. Cell nuclei were stained with DAPI. Fluorescence images were obtained using a fluorescence or confocal microscope, and the fluorescence intensity of iNOS was quantified using ImageJ software.

### Evaluation of cell migration and angiogenesis

To investigate the effects of different materials on cell migration and angiogenesis-related behaviors, HUVECs and HOK were used for *in vitro* functional assays. For the wound healing assay, HUVECs or HOK were seeded in six-well plates and cultured until nearly 100% confluence. A straight scratch was created across the cell monolayer using a sterile pipette tip, followed by gentle washing with PBS to remove detached cells. Cells were then cultured in serum-free medium containing different materials, and images of the wound area were captured at designated time points. The wound closure rate was quantified using ImageJ to evaluate cell migration capability.

Cell migration was further assessed using a Transwell assay. Cell suspensions in serum-free medium were added to the upper chamber, while the lower chamber was filled with medium containing 10% FBS as a chemoattractant, along with different materials. After incubation for 24 h, non-migrated cells on the upper surface were removed. Cells that had migrated to the lower membrane were fixed with 4% paraformaldehyde and stained with crystal violet. Multiple random fields were imaged under a microscope, and the number of migrated cells was quantified.

For the tube formation assay, pre-cooled Matrigel was added to 96-well plates and incubated at 37°C to allow gelation. HUVECs were then seeded onto the Matrigel surface and cultured with medium containing different materials. After 6 h of incubation, capillary-like structures were observed and imaged using an inverted microscope.

To further evaluate angiogenesis-related molecular expression, the level of vascular endothelial growth factor (VEGF) in HUVECs was analyzed. After treatment with different materials, cells were fixed with 4% paraformaldehyde, permeabilized with 0.1% Triton X-100 and blocked with 5% bovine serum albumin. Samples were then incubated with primary antibody against VEGF at 4°C overnight, followed by incubation with a fluorescently labeled secondary antibody for 1 h. Nuclei were stained with DAPI. Fluorescence images were acquired using a fluorescence or confocal microscope, and fluorescence intensity was quantified using ImageJ.

### Establishment of rat oral ulcer model and *in vivo* therapeutic evaluation

To evaluate the therapeutic efficacy of different materials in oral mucosal repair *in vivo*, healthy male Sprague–Dawley (SD) rats were used to establish an oral ulcer model. All animal experiments were approved by the Institutional Animal Care and Use Committee of Shanxi Medical University with approval number KQDW-2024-004. The oral ulcer model was induced using an acetic acid chemical injury method. The acetic acid-induced oral ulcer model is a widely used acute mucosal injury model with good reproducibility and controllable inflammatory responses, making it suitable for evaluating local therapeutic materials and early-stage mucosal repair efficacy. However, this model primarily reflects chemically induced acute inflammatory injury and does not fully recapitulate the complex immune dysregulation, recurrent characteristics or multifactorial etiology observed in clinical recurrent aphthous ulcers and other chronic ulcerative disorders. Under anesthesia, a filter paper disk with a diameter of 3 mm soaked in 50% acetic acid solution was applied to the buccal mucosa of rats for ∼60 s, followed by thorough rinsing with saline to remove residual acid. To enhance model stability, the procedure was repeated after 24 h to induce ulcer lesions with evident epithelial disruption and inflammatory response.

After successful model establishment, the animals were randomly divided into four groups and materials of identical size were applied to cover the ulcer area. During the treatment period, standardized photographs of the ulcer regions were taken at predetermined time points. The ulcer area was measured using ImageJ, and the relative healing rate was calculated to quantitatively evaluate the therapeutic effects of different treatments.

At the experimental endpoint, animals were sacrificed and ulcer tissues were harvested, fixed in 4% paraformaldehyde, embedded in paraffin and sectioned. Hematoxylin and eosin (H&E) staining was performed to assess epithelial integrity, inflammatory cell infiltration and tissue structural recovery. Masson staining was used to evaluate collagen deposition and alignment, thereby analyzing tissue reconstruction and extracellular matrix remodeling.

Neovascularization and vascular maturation were evaluated by IF staining of CD31 and *α*-SMA. After deparaffinization, antigen retrieval and blocking, tissue sections were incubated with primary antibodies against CD31 and *α*-SMA, followed by fluorescent secondary antibodies. Vascular density and smooth muscle layer formation were observed under a fluorescence microscope to assess angiogenesis and vessel maturation.

In addition, immunohistochemical (IHC) staining was performed to evaluate the local inflammatory microenvironment by detecting the expression of TNF-*α* and IL-10 in ulcer tissues. After deparaffinization, antigen retrieval and blocking, sections were incubated with the corresponding primary antibodies and visualized using DAB, followed by hematoxylin counterstaining. The staining intensity was observed under a light microscope and compared among groups to assess inflammatory status and immunomodulatory effects. For IF and IHC quantification, at least five independent biological samples were analyzed for each group. Five random fields from each section were selected for quantitative analysis using ImageJ software under identical imaging parameters.

### Statistical analysis

All experiments were performed at least three times independently, and data are presented as mean ± standard deviation (SD). Statistical analysis was conducted using GraphPad Prism. Comparisons between two groups were performed using an unpaired Student’s *t*-test. Multiple group comparisons were conducted using one-way analysis of variance (ANOVA) followed by Tukey’s multiple comparisons test. For experiments involving time-dependent variables, two-way ANOVA was applied. Statistical significance was defined as *P *< 0.05, *P < 0.05, **P < 0.01, ***P < 0.001 and ****P < 0.0001.

## Results and discussion

### Synthesis and characterization of BZ

BZ was synthesized via a one-step self-assembly strategy, in which berberine was simultaneously introduced during the coordination of Zn^2+^ with 2-methylimidazole. This *in situ* encapsulation approach enables the concurrent formation of the MOF structure and drug loading, thereby ensuring a homogeneous distribution of berberine within the ZIF nanostructure rather than simple surface adsorption.

SEM analysis revealed that ZIF exhibited a typical rhombic dodecahedral morphology with a uniform particle size distribution (Supplementary Figure S1). In comparison, BZ retained the well-defined crystalline morphology while showing a slightly increased particle size (Figure 2A). Notably, no crystalline features corresponding to berberine were observed, further confirming that berberine was incorporated within the ZIF framework in an embedded form. UV-vis spectroscopy (Figure 2B) showed that BZ displayed the characteristic absorption peaks of berberine at 345 nm and 425 nm, indicating successful encapsulation of the drug within the ZIF framework. It is noteworthy that the absorbance intensity of these peaks in BZ was reduced compared with free berberine, which may be attributed to the restricted molecular mobility of berberine within the MOF pores or alterations in its microenvironment. Meanwhile, the intrinsic absorption peak of ZIF at ∼240 nm was well preserved, suggesting that the structural integrity of the framework remained intact after drug loading. In addition, slight shifts or peak broadening of the characteristic berberine signals were observed in BZ, implying the presence of weak interactions between berberine molecules and the ZIF framework.

**Figure 2:**
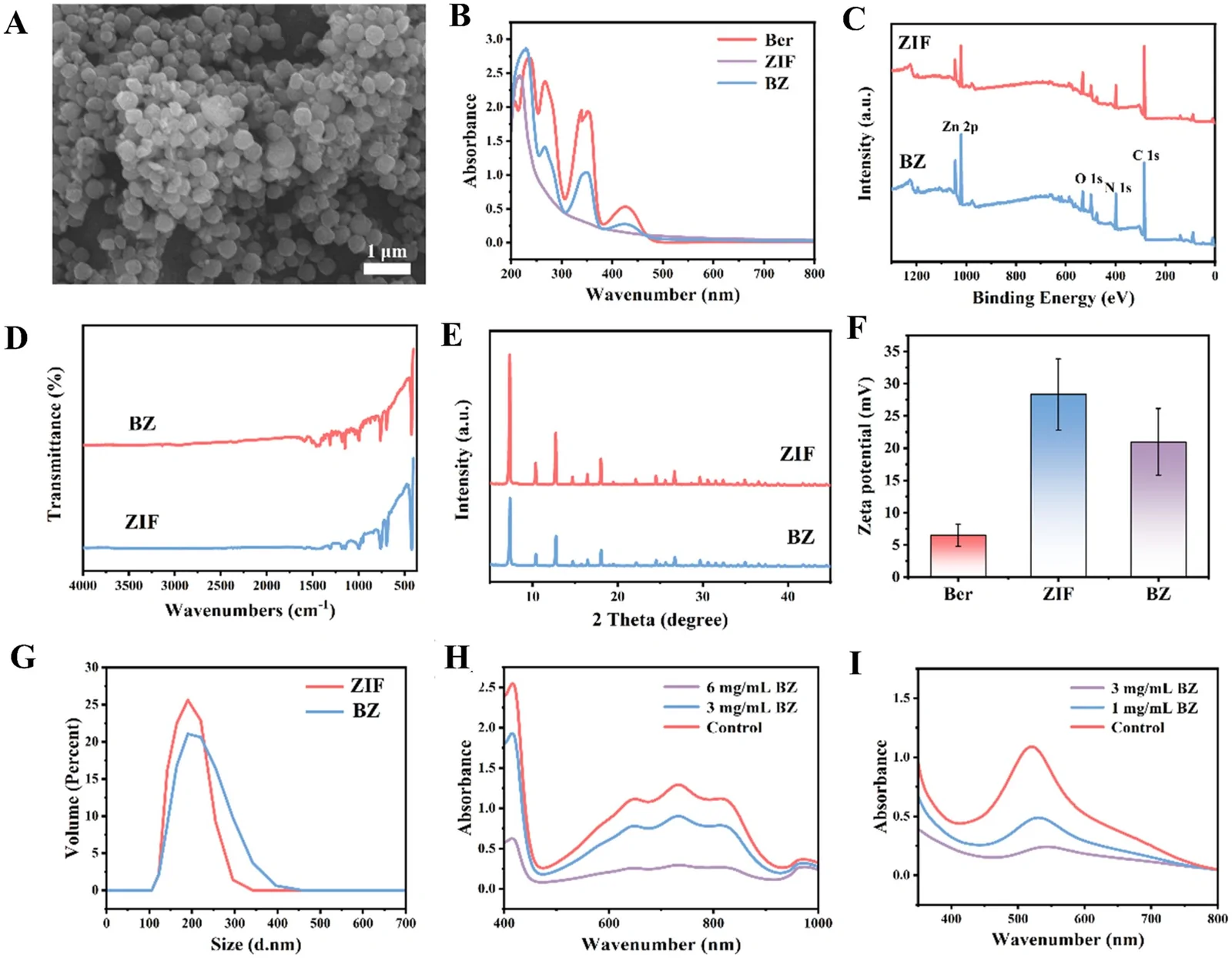
Physicochemical characterization and antioxidant activity of BZ nanoparticles. (**A**) SEM images of BZ. (**B**) UV-vis absorption spectra of berberine, ZIF and BZ. (**C**) XPS survey spectra of ZIF and BZ. (**D**) FT-IR spectra of ZIF and BZ. (**E**) XRD patterns of ZIF and BZ. (**F**) Zeta potential of berberine, ZIF and BZ. (**G**) Hydrodynamic size distribution of ZIF and BZ measured by DLS. (**H**) ABTS•^+^ radical scavenging activity of BZ at different concentrations. (**I**) DPPH• radical scavenging activity of BZ at different concentrations. Data are presented as mean ± SD (*n* = 3 independent experiments).

XPS analysis demonstrated that both ZIF and BZ were primarily composed of Zn, C, O and N elements (Figure 2C). Compared with ZIF, BZ exhibited significantly enhanced C 1s and N 1s signal intensities, indicating the successful incorporation of berberine molecules rich in aromatic heterocyclic structures into the ZIF system. The increased O 1s signal further supported the introduction of oxygen-containing functional groups from berberine. The Zn 2p peaks remained clearly detectable in both samples, confirming that the ZIF framework maintained its structural integrity after drug loading. Moreover, BZ displayed a greater number of characteristic peaks, which can be attributed to the additional electronic binding energy features introduced by berberine, along with the increased content of C, O and N elements, resulting in enhanced signal intensity and peak complexity in the XPS spectra. FT-IR analysis (Figure 2D) revealed that both samples exhibited a characteristic stretching vibration peak of the Zn-N bond at around 500 cm^−1^, indicating that the fundamental framework of ZIF remained intact after berberine encapsulation. Compared with ZIF, BZ displayed a series of new and intensified absorption bands in the range of 1000–1600 cm^−1^. Specifically, the peaks at 1450–1600 cm^−1^ correspond to the C = C stretching vibrations of the aromatic rings in berberine, while those at 1100–1250 cm^−1^ are attributed to the C–O stretching vibrations of methoxy groups. These characteristic functional group signals confirm the successful incorporation of berberine into the ZIF framework.

XRD patterns (Figure 2E) showed that BZ retained the characteristic diffraction peaks of ZIF, indicating that the introduction of berberine did not disrupt the crystalline structure of the MOF. Notably, no diffraction peaks corresponding to crystalline berberine were observed in BZ, suggesting that berberine exists in an amorphous or molecularly dispersed state within the ZIF pores. The surface charge properties were characterized by zeta potential analysis (Figure 2F). Free berberine exhibited a relatively low positive zeta potential of ∼+6.5 ± 1.7 mV. In contrast, ZIF showed a significantly higher positive potential of +28.3 ± 5.5 mV, which can be attributed to the exposed metal nodes and protonated imidazole groups on the framework surface. After drug loading, the zeta potential of BZ decreased to +20.9 ± 5.1 mV. This reduction indicates that the incorporation of berberine partially shields the positively charged sites on the ZIF surface, leading to a decrease in interfacial charge density. Nevertheless, BZ retained an overall positive surface charge, suggesting that the ZIF framework still dominates the interfacial properties, which is beneficial for interactions with negatively charged oral mucosa and bacterial membranes. DLS analysis (Figure 2G) further characterized the hydrodynamic size distribution of the nanoparticles in aqueous solution. ZIF-8 displayed a relatively narrow size distribution with an average hydrodynamic diameter of 226.3 nm, while BZ possessed a significantly larger average particle size of 271.7 nm, presenting an obviously increased hydrodynamic diameter compared with pure ZIF-8. This increase is consistent with the expansion of the crystal structure and the enhanced hydration layer resulting from the incorporation of berberine into the ZIF framework.

To evaluate the antioxidant capacity of BZ, two classical radical scavenging models, ABTS•^+^ and DPPH• radical scavenging assays, were employed for systematic assessment (Figure 2H and I). The results demonstrated that BZ exhibited pronounced free radical scavenging activity in both systems, with a clear concentration-dependent enhancement. Specifically, BZ at 6 mg/mL showed significantly higher ABTS•^+^ radical scavenging efficiency than that at 3 mg/mL, while BZ at 3 mg/mL also exhibited stronger DPPH• radical scavenging activity compared with 1 mg/mL. These findings indicate that the antioxidant capacity of BZ is concentration-dependent and support its strong free radical scavenging potential. From a mechanistic perspective, the antioxidant activity of BZ primarily originates from the conjugated aromatic structure of berberine, which provides effective electron-donating capability. This enables electron transfer or hydrogen atom transfer reactions with reactive radicals, thereby neutralizing them. Meanwhile, the porous ZIF framework serves as a stable carrier for berberine and offers a large specific surface area and accessible reaction interfaces in aqueous environments, facilitating the interaction between berberine and radical species and thereby enhancing the overall antioxidant efficiency. It should be noted that ZIF itself typically exhibits negligible radical scavenging activity; therefore, the observed antioxidant performance of BZ can be mainly attributed to the incorporation of berberine.

Taken together, BZ was successfully constructed via a one-step self-assembly strategy. Comprehensive characterization confirmed that berberine was effectively encapsulated within the ZIF framework while maintaining well-defined crystalline structure and stable nanoscale morphology. BZ exhibited an appropriate size distribution in aqueous solution along with an overall positive surface charge, which not only contributes to its colloidal stability but may also enhance its interactions with negatively charged oral mucosa and bacterial membranes. In addition, radical scavenging assays demonstrated that BZ possesses significant and concentration-dependent antioxidant activity, confirming that the incorporation of berberine into the MOF matrix endows the material with desirable antioxidative functionality. Overall, the constructed BZ combines structural stability, positive surface characteristics and antioxidant activity, enabling effective interaction with negatively charged oral interfaces and modulation of oxidative stress, thereby providing a solid foundation for its application in oral therapeutic systems.

### Synthesis and characterization of BZB

The stable incorporation of BZ within the BC network is primarily attributed to multiple interfacial interactions. First, the abundant hydroxyl groups on the surface of BC nanofibers can form a dense hydrogen bonding network with the N and O atoms in the ZIF framework and berberine molecules. In addition, weak coordination interactions may occur between these functional groups and partially exposed Zn sites. Second, under aqueous conditions, BZ exhibits a distinctly positive surface potential, whereas BC typically carries a slightly negative surface charge. This electrostatic attraction further enhances the interfacial binding stability between BZ and the BC matrix. Moreover, the unique three-dimensional porous nanofibrous architecture of BC provides a pronounced physical confinement effect on BZ nanoparticles, effectively suppressing particle aggregation and promoting their uniform distribution within the matrix. The synergistic contribution of these interactions enables the stable anchoring of BZ within the BC substrate.

SEM observations further revealed the distinct Janus asymmetric architecture of BC. One side exhibited a highly porous and loosely interconnected network (Figure 3A), whereas the opposite side consisted of densely packed and tightly entangled nanofibers (Figure 3B). In the BZB composite system, BZ nanoparticles were clearly observed to be predominantly anchored and enriched within the porous side. The larger pore size and higher specific surface area of this region provide sufficient physical space and abundant binding sites for nanoparticle loading (Figure 3C). In contrast, the dense layer maintained a relatively smooth and low porosity structure (Figure 3D), which is advantageous for forming a stable adhesive interface in subsequent applications. To further evaluate the structural stability of BZB under oral-mimicking conditions, SEM analysis was performed after incubation in artificial saliva (Supplementary Figure S2). The results showed that the composite maintained its characteristic Janus architecture, with both the porous drug-loading layer and the dense adhesive layer remaining clearly identifiable. No obvious structural collapse, fiber fragmentation or nanoparticle aggregation was observed after salivary exposure. The nanofibrous network remained highly interconnected and structurally intact, indicating that the BC matrix effectively protects the composite from salivary erosion. These findings suggest that BZB possesses excellent morphological stability in artificial saliva, supporting its potential for prolonged retention and sustained local therapy in the dynamic oral environment.

**Figure 3:**
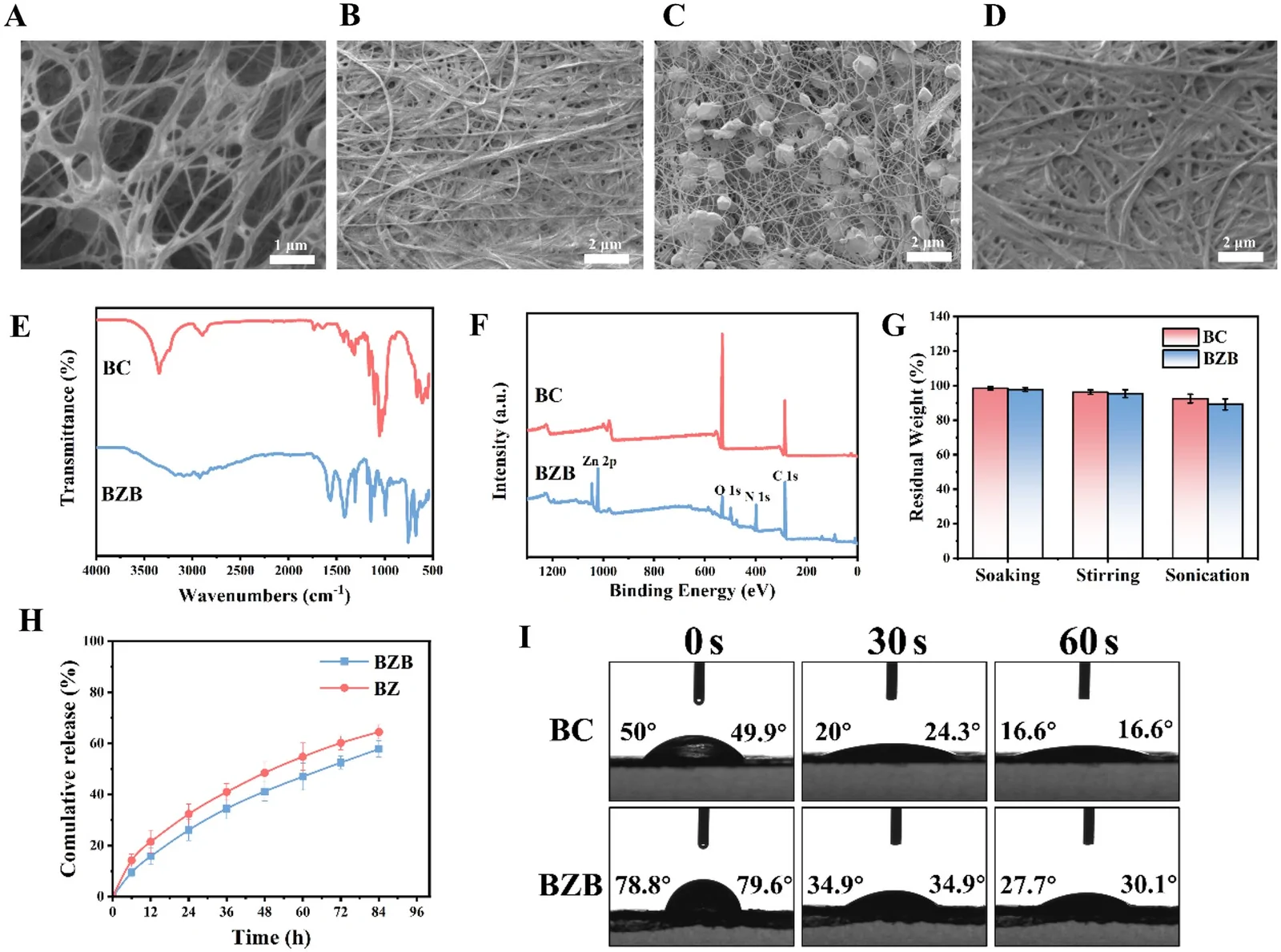
Structural characterization, interfacial interactions and functional properties of BZB. SEM images showing the Janus asymmetric structure of BC with (**A**) a porous layer and (**B**) a dense layer. SEM images of BZB, illustrating the preferential distribution of BZ nanoparticles within the (**C**) porous layer and the compact structure of (**D**) the dense layer. (**E**) FT-IR spectra of BC and BZB. (**F**) XPS spectra of BC and BZB. (**G**) Structural persistence under oral-mimicking conditions of BC and BZB under static immersion, magnetic stirring and ultrasonic treatment. (**H**) *In vitro* cumulative release profiles of berberine from BZ and BZB. (**I**) Time-dependent contact angle measurements of BC and BZB. Data are presented as mean ± SD (*n* = 3 independent experiments).

FT-IR analysis further confirmed the effective integration of BZ within the BC matrix (Figure 3E). BZB retained the characteristic absorption bands of BC, including the broad O-H stretching vibration at ∼3340 cm^−1^, the C–H stretching at around 2900 cm^−1^ and the C–O–C glycosidic bond vibrations in the range of 1000–1160 cm^−1^, indicating that the BC structure remained intact during composite formation. Meanwhile, new characteristic peaks appeared in the range of 750–600 cm^−1^, corresponding to the out-of-plane bending vibration of the imidazole ring in ZIF. In addition, an intensified absorption band in the region of 1580–1450 cm^−1^ was observed, which can be attributed to the overlapping contributions of aromatic ring vibrations from berberine and the C = N stretching vibration of the imidazole ring in ZIF, confirming the successful incorporation of the functional components. Notably, the significant broadening and slight red shift of the O-H stretching band in BZB suggest the formation of strong hydrogen bonding networks or coordination interactions between the hydroxyl groups of BC and BZ. XPS spectra (Figure 3F) showed that BC exhibited only the characteristic peaks of C 1s and O 1s, consistent with its polysaccharide composition. In contrast, BZB displayed distinct Zn 2p doublet peaks along with a detectable N 1s signal, which can be attributed to the zinc nodes in ZIF and the nitrogen atoms from both imidazole ligands and berberine molecules. These pronounced changes in elemental composition provide direct surface chemical evidence for the successful construction of BZB.

To evaluate whether the patch could maintain sufficient structural integrity during the oral ulcer healing period, BC and BZB were exposed to oral environment-mimicking conditions in both PBS and artificial saliva, including static incubation, magnetic stirring and ultrasonic disturbance (Figure 3G;  Supplementary Figure S3). Since oral ulcer repair is typically completed within ∼1 week, maintaining temporary structural persistence rather than rapid degradation is considered beneficial for sustained local therapy and protection of the lesion site. The resistance to degradation was quantitatively assessed by measuring the RWR after treatment. Under static incubation conditions, BC and BZB exhibited excellent stability in both PBS and artificial saliva. These results indicate that the incorporation of BZ did not compromise the integrity of the BC network and that no substantial component loss occurred under quiescent conditions. The slightly lower RWR observed in artificial saliva may be attributed to the more complex ionic composition and salivary constituents, which can enhance surface erosion and ion exchange processes. Under magnetic stirring conditions, both materials exhibited moderate weight loss due to continuous mechanical shear. The comparable retention of BZB suggests that the hydrogen bonding, electrostatic interactions and possible coordination interactions between BZ and the BC matrix effectively restrict nanoparticle detachment and preserve the structural integrity of the composite under dynamic conditions. To further evaluate extreme mechanical stability, the samples were subjected to ultrasonic treatment. The slightly lower RWR of BZB may result from partial detachment of surface-exposed ZIF particles caused by cavitation effects, as well as a modest reduction in network flexibility due to the incorporation of rigid inorganic components. Nevertheless, both materials maintained high residual mass, demonstrating excellent resistance to mechanical disruption. Overall, BZB exhibited favorable structural persistence in both PBS and artificial saliva. Although artificial saliva caused slightly greater mass loss than PBS, the composite retained the majority of its original mass even under dynamic and ultrasonic conditions. These findings indicate that the BC matrix effectively confines BZ nanoparticles within its network and enables the composite to withstand salivary exposure and substantial mechanical challenges, thereby supporting prolonged retention and sustained therapeutic activity in the oral cavity.

To enable quantitative determination of berberine in all subsequent analyses, including drug loading, encapsulation efficiency, berberine content in BZB and release studies, a standard calibration curve was established using UV-vis spectroscopy (Supplementary Figure S4). Berberine was dissolved in PBS to prepare standard solutions with concentrations ranging from 2 to 60 μg/mL. Based on full wavelength scanning, the characteristic absorption peak was identified at 345 nm (Figure 2B). The absorbance at this wavelength was plotted against concentration, showing a good linear relationship within the range of 2–60 μg/mL, with a regression equation of *y* = 0.02301*x* + 0.01587 and a correlation coefficient *R*^2^ = 0.994. Quantitative UV-vis analysis demonstrated efficient encapsulation of berberine within the ZIF framework. The EE and DLC of BZ were determined to be 73.4 ± 3.6% and 11.7 ± 1.8%, respectively. These results indicate that the one-step *in situ* self-assembly process enabled effective incorporation of berberine into the ZIF nanostructure while maintaining the structural integrity of the framework. Furthermore, the actual berberine content in the optimized BZB formulation (BZB2), determined after nanoparticle incorporation and washing, was 2.85 ± 0.3 mg berberine per gram of dry patch, providing a quantitative basis for subsequent release and biological evaluations. The measured berberine content in BZB was lower than the theoretical value calculated from the DLC of BZ, which may be attributed to the removal of loosely attached nanoparticles during the infiltration and subsequent washing procedures.

As shown in Supplementary Figure S5, both BC and BZB patches exhibited robust adhesive performance following artificial saliva soaking, stirring and sonication treatments, with no apparent loss of adhesion strength. Quantitative analysis demonstrated no statistically significant differences either among the various treatment conditions or between the BC and BZB groups, suggesting that the incorporation of Ber@ZIF did not adversely affect the adhesive capability of the BC substrate. These results indicate that the patches possess strong resistance to salivary flushing and mechanical disturbances, while maintaining stable wet adhesion and interfacial integrity, thereby supporting their suitability for prolonged retention in the dynamic oral environment.

The calibration curve was subsequently used to calculate the cumulative release of berberine in the *in vitro* release studies. As shown in Figure 3H, BZ exhibited an initial rapid release followed by a sustained release phase. In contrast, BZB demonstrated a more controlled release profile. The BC membrane acted as an additional diffusion barrier, effectively reducing the initial burst release and slowing the subsequent release rate. These results confirm the critical role of BC in prolonging drug delivery and improving release controllability.

To further investigate surface wettability, the dynamic contact angle evolution was monitored (Figure 3I). At the initial contact stage (0 s), BC exhibited rapid wetting behavior due to the abundance of hydrophilic hydroxyl groups on the nanofiber surface. As time progressed to 60 s, the liquid droplet gradually penetrated into the porous network driven by capillary forces, leading to a sharp decrease in the contact angle to ∼16.6°, indicating its strong water absorption and swelling capability. In contrast, BZB displayed a moderately increased hydrophobic characteristic. At the same time point of 60 s, the contact angle remained around 30°, which can be attributed to the successful incorporation of BZ that partially masked the hydrophilic sites on the BC surface and filled portions of the porous network, thereby hindering rapid capillary infiltration of water molecules. Notably, despite the increase in contact angle, BZB still maintained favorable hydrophilicity. This suggests that the modification strategy introduces a balanced interfacial property, providing a mild hydrophobic barrier to regulate drug release kinetics without compromising the essential wettability required for biomedical applications.

In summary, a Janus-structured composite patch BZB was successfully developed. These findings indicate that the BZB system integrates favorable structural integrity under oral-mimicking conditions, sustained drug release capability and appropriate interfacial wettability, thereby establishing a solid material basis for its application as a localized oral therapeutic patch. Notably, oral ulcer treatment generally requires only short-term intervention during the acute inflammatory and re-epithelialization stages, which are typically completed within ∼5–10 days. Under such conditions, maintaining temporary structural stability and wet adhesion throughout the healing window is more beneficial than rapid bulk degradation, as it helps preserve local drug retention and lesion protection in the dynamic oral environment. In this system, the BC matrix serves as a transient protective and drug-retaining platform, while the therapeutically active BZ component gradually releases berberine and Zn^2+^ in response to the inflammatory microenvironment, thereby continuously supporting ulcer repair.

### *In vitro* antibacterial activity of BZB

Oral ulcer lesions are continuously exposed to a high load of oral microorganisms. Bacterial colonization not only exacerbates local inflammatory responses but also disrupts epithelial cell migration and reepithelialization, thereby significantly delaying ulcer healing [42, 43]. Therefore, an ideal therapeutic patch for oral ulcers should not only possess strong adhesion and efficient drug delivery capability but also effectively inhibit pathogenic bacterial growth to interrupt the vicious cycle of infection, inflammation and tissue damage [44]. Based on this rationale, the antibacterial activity of BZB against representative Gram-positive *S.aureus* and Gram-negative *E.coli* was systematically evaluated.

Colony counting results (Figure 4A and B) showed that both the Control and BC groups formed abundant bacterial colonies, with no significant difference between them, indicating that BC itself does not exhibit intrinsic antibacterial activity. In contrast, all BZB-treated groups demonstrated pronounced antibacterial effects, with a clear dose-dependent trend as the BZ loading increased. Specifically, BZB1 partially inhibited bacterial growth, whereas BZB2 and BZB3 groups exhibited a significant reduction in colony numbers. Notably, the highest concentration group almost completely suppressed the survival of both bacterial strains, demonstrating potent broad-spectrum antibacterial activity.

**Figure 4:**
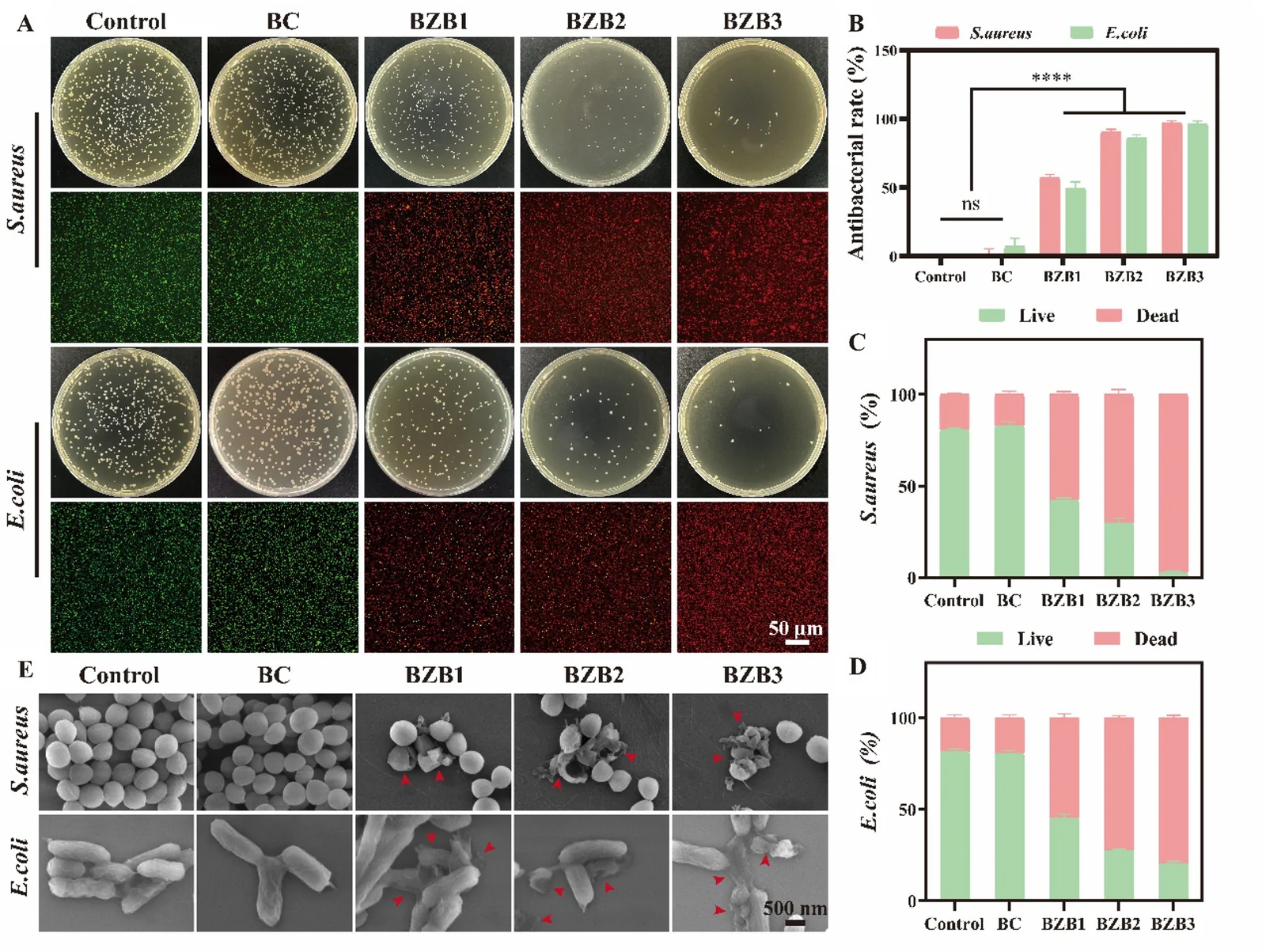
*In vitro* antibacterial activity of BZB. (**A**) Representative images of bacterial colonies and live/dead fluorescence staining under different treatments. (**B**) Quantitative analysis of antibacterial rates based on colony counting. Quantitative analysis of live/dead staining for (**C**) S.aureus and (**D**) *E.coli.* (**E**) SEM images showing morphological changes of bacteria after different treatments. Data are presented as mean ± SD (*n* = 3 independent experiments). Statistical analysis was performed using one-way ANOVA followed by Tukey’s multiple comparisons test. ns, not significant. *P < 0.05, **P < 0.01, ***P < 0.001 and ****P < 0.0001.

Live/dead fluorescence staining further confirmed this trend (Figure 4A, C and D). In the Control and BC groups, bacteria predominantly exhibited green fluorescence, indicating intact cell membranes and high viability. In contrast, the proportion of red fluorescence, representing dead bacteria, increased progressively with higher BZ content in the BZB groups. Particularly in the BZB3 group, most bacteria exhibited loss of membrane integrity and appeared in a dead state, demonstrating that the system effectively disrupts bacterial cellular structures.

SEM observations further elucidated the antibacterial mechanism (Figure 4E). In the Control and BC groups, both *S.aureus* and *E.coli* exhibited smooth surfaces and intact morphology, maintaining their typical spherical or rod-shaped structures. In contrast, after treatment with BZB, bacteria displayed pronounced morphological damage, including surface wrinkling, collapse and rupture, with evident leakage of intracellular contents. This structural disruption became more severe with increasing BZ concentration, indicating that the material induces substantial membrane damage through synergistic physical and chemical interactions.

The pronounced antibacterial activity can be attributed to the multifaceted mechanisms of BZ. On one hand, ZIF gradually releases Zn^2+^ under the mildly acidic bacterial microenvironment, which can interact with membrane proteins and enzymatic systems, thereby disrupting membrane permeability and inhibiting metabolic activity [45]. On the other hand, berberine, as a natural cationic antibacterial molecule, can insert into bacterial membranes and interfere with DNA and protein synthesis. Meanwhile, the three-dimensional network structure of BC enables stable anchoring of BZ at the interface, creating a localized microenvironment with sustained and effective antibacterial action.

Collectively, BZB exhibits concentration-dependent broad-spectrum bactericidal activity against *S.aureus* and *E.coli*, providing a solid functional basis for its application in the treatment of infection-associated oral ulcers.

### Biocompatibility of BZB

For local patches intended for oral ulcer treatment, favorable biosafety is a fundamental prerequisite for achieving prolonged adhesion and effective tissue repair [46]. To evaluate the cytocompatibility and hemocompatibility of BZB, HOK were employed as the model cell line, and cell viability and cytotoxicity were assessed at multiple time points. CCK-8 results (Figure 5B) showed that at Day 1, Day 3 and Day 5, there was no significant difference in cell viability between the Control and BC groups, indicating that BC itself possesses excellent cytocompatibility without inducing adverse cellular effects. With the incorporation of BZ, the BZB1 and BZB2 groups exhibited higher cell viability than the BC group at all time points, with a clear concentration-dependent enhancement. This effect can be primarily attributed to the gradual release of Zn^2+^ from ZIF under culture conditions. As an essential trace element for various cellular enzymes and transcription factors, Zn^2+^ promotes the expression of genes associated with cell proliferation and migration, thereby enhancing the viability and growth state of HOK. In contrast, the BZB3 group exhibited a decrease in cell viability compared with BZB2, likely due to the excessive loading of BZ. The elevated levels of Zn^2+^ and berberine may induce mild cytotoxicity, partially offsetting their proliferative benefits and reflecting a typical dose-dependent window effect.

**Figure 5:**
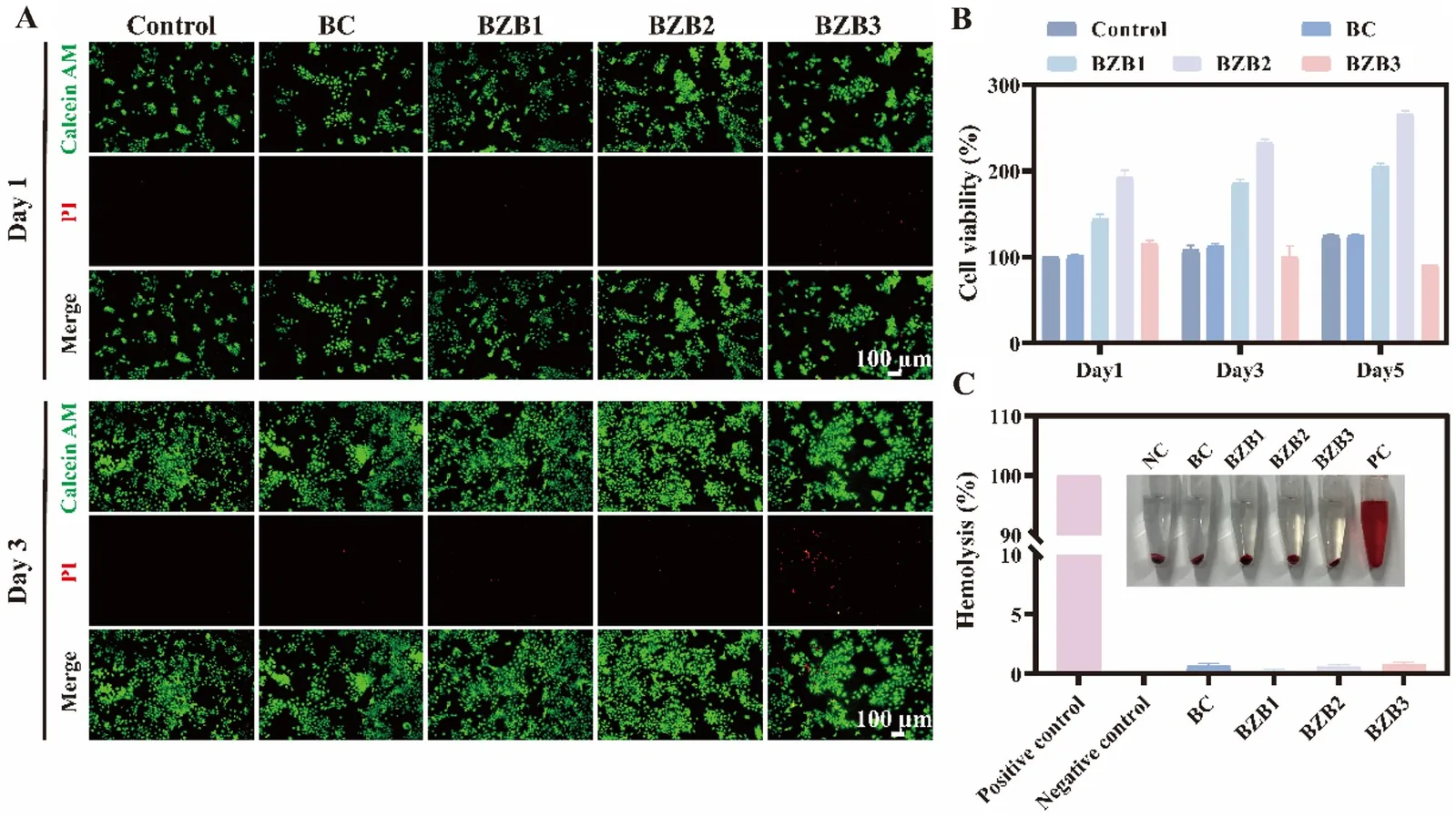
Biocompatibility evaluation of BZB. (**A**) Live/dead fluorescence staining of HOK under different treatments. (**B**) Cell viability of HOK assessed by CCK-8 assay at different time points. (**C**) Hemolysis assay of BC and BZB with different loading concentrations. Data are presented as mean ± SD (*n* = 3 independent experiments). Statistical analysis was performed using two-way ANOVA with repeated measures followed by Tukey’s multiple comparisons test. ns, not significant. *P < 0.05, **P < 0.01, ***P < 0.001 and ****P < 0.0001.

Live/dead staining results of HOK were highly consistent with the CCK-8 results (Figure 5A). The BZB1 and BZB2 groups displayed a noticeably higher cell density than the Control and BC groups, with well-spread cellular morphology, indicating enhanced cell survival and proliferation. Although the BZB3 group still predominantly exhibited viable cells, a small number of red-stained dead cells were observed, suggesting that excessive BZ loading may induce slight cytotoxic effects.

In addition, a hemolysis assay was performed to further evaluate the hemocompatibility of the materials (Figure 5C). The results showed that BC and all BZB groups exhibited hemolysis ratios well below the clinically acceptable threshold, with no significant differences among different BZ loading levels. These findings indicate that the system does not induce appreciable damage to erythrocyte membranes and possesses favorable blood compatibility.

Taken together, the results from cell viability assays, live/dead staining and hemolysis analysis demonstrate that BZB exhibits excellent biocompatibility within an appropriate loading range. More importantly, by precisely regulating the incorporation level of BZ within the BC matrix, it is possible to achieve an effective balance between enhanced antibacterial activity and minimal cytotoxicity. Although increasing BZ content contributes to improved antibacterial performance, excessive loading may lead to cellular stress due to elevated Zn^2+^ release and nanoparticle accumulation, thereby reducing cell viability. Based on this typical dose-dependent window, BZB2, hereafter referred to as BZB, was selected as the representative formulation for subsequent *in vitro* and *in vivo* evaluations, as it provides an optimal balance between antibacterial efficacy and cellular safety. This balance is of critical importance for promoting tissue repair in oral ulcer lesions.

### BZB alleviates oxidative stress and preserves cell viability under oxidative damage conditions

To mimic the persistent oxidative stress microenvironment of oral ulcers, H_2_O_2_ was used to induce excessive intracellular ROS generation in HUVECs, and the protective effects of different materials against oxidative damage were evaluated. Intracellular ROS levels were detected using the DCFH-DA probe. As shown in Figure 6A and B, cells in the untreated Blank group maintained low basal fluorescence intensity, whereas the H_2_O_2_-treated Control and BC groups exhibited markedly increased green fluorescence signals, indicating substantial intracellular ROS accumulation. In contrast, the BZ group showed a significant reduction in ROS fluorescence intensity, confirming its effective intracellular antioxidant activity. Notably, the BZB group demonstrated a further pronounced decrease in ROS levels, approaching those of the Blank group, indicating the most effective attenuation of oxidative stress.

**Figure 6:**
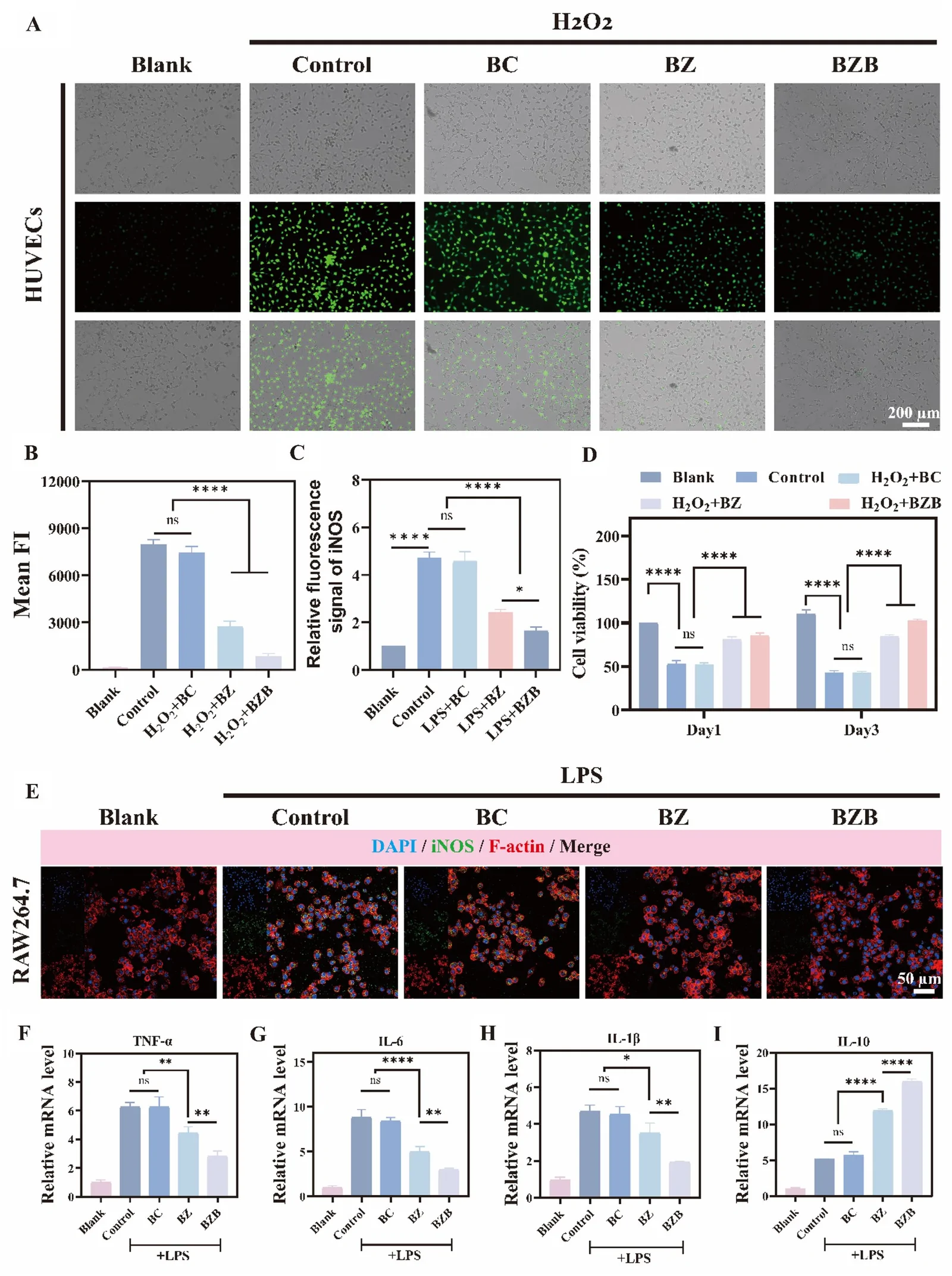
BZB alleviates oxidative stress and modulates the inflammatory microenvironment. (**A**) Intracellular ROS levels visualized by DCFH-DA staining. (**B**) Quantification of ROS fluorescence intensity. (**C**) Quantitative analysis of IF staining of iNOS expression. (**D**) Cell viability of HUVECs under H_2_O_2_-induced oxidative stress. (**E**) IF staining of iNOS expression. RT-qPCR analysis of inflammatory cytokine expression in RAW264.7 cells, including (**F**) TNF-α, (**G**) IL-6, (**H**) IL-1β and (**I**) IL-10. Data are presented as mean ± SD (*n* = 3 independent experiments). Statistical analysis was performed using one-way ANOVA followed by Tukey’s multiple comparisons test. ns, not significant. *P < 0.05, **P < 0.01, ***P < 0.001 and ****P < 0.0001.

This enhanced effect arises not only from the intrinsic antioxidant capacity of BZ but also from the interfacial characteristics of BC. Although BC itself lacks direct chemical antioxidant activity, its extracellular matrix-like nanofibrous network provides a stable adhesion microenvironment for cells and localizes BZ at the cell-material interface, thereby increasing its effective concentration in the cellular vicinity and amplifying its antioxidant effect. As a result, BZB suppresses intracellular ROS accumulation more efficiently than BZ alone.

Cell viability assays further supported these findings (Figure 6D). Under H_2_O_2_-induced oxidative damage, the Control and BC groups exhibited significantly reduced cell viability at both Day 1 and Day 3, indicating that the BC matrix alone is insufficient to counteract oxidative stress-induced injury. Treatment with BZ led to a marked recovery in cell viability at both time points, demonstrating its protective role against ROS-mediated cellular damage. Importantly, the BZB group exhibited the highest cell survival rates at both Day 1 and Day 3, significantly outperforming the BZ group. This suggests that the composite system not only effectively scavenges ROS but also better preserves cellular function and proliferative capacity under sustained oxidative stress conditions.

Collectively, the superior antioxidant and cytoprotective performance of BZB can be attributed to its ability to reconstruct the cellular redox microenvironment. Zn^2+^ released gradually from ZIF serves as a critical cofactor for various antioxidant enzymes, contributing to the maintenance of intracellular redox homeostasis, while berberine stabilizes mitochondrial function and suppresses excessive ROS generation [47, 48]. When integrated into the BC hydrogel, the release behavior transitions from a rapid burst to a controlled and sustained mode, enabling continuous antioxidant protection throughout the repair process. Therefore, compared with BZ alone, BZB not only achieves more efficient ROS scavenging but also maintains long-term cell viability under oxidative stress, thereby providing a more favorable microenvironment for epithelial regeneration and angiogenesis in oral ulcer healing.

### Regulation of the inflammatory microenvironment by BZB

Inflammatory response is a central driver of persistent deterioration and delayed healing in oral ulcers. Sustained overexpression of pro-inflammatory cytokines not only directly damages cells but also amplifies oxidative stress and disrupts regeneration-related signaling pathways [49–51]. Therefore, effective suppression of local pro-inflammatory factors and restoration of pro-regenerative cytokine balance are critical prerequisites for promoting ulcer healing.

In this study, RT-qPCR was employed to systematically evaluate the expression of inflammation-related genes in RAW264.7 cells treated with different materials. Compared with the Control group, the BC group did not exhibit a significant inhibitory effect on pro-inflammatory cytokine expression, indicating that the BC scaffold primarily provides structural support without intrinsic anti-inflammatory activity. In contrast, BZ treatment significantly downregulated the mRNA expression levels of TNF-*α* (Figure 6F), IL-6 (Figure 6G) and IL-1*β* (Figure 6H), while upregulating IL-10 expression (Figure 6I), demonstrating its inherent anti-inflammatory properties. Notably, the BZB group exhibited the most pronounced anti-inflammatory effects. The expression levels of TNF-*α*, IL-6 and IL-1*β* were markedly lower than those in the BZ group, whereas IL-10 expression was further elevated. These findings indicate that integrating BZ into the BC matrix not only preserves its anti-inflammatory activity but also enhances its therapeutic efficacy by improving local retention and delivery stability, enabling sustained action within the cellular microenvironment.

To further validate the anti-inflammatory effect at the protein level, IF staining of the pro-inflammatory marker iNOS was performed in RAW264.7 cells (Figure 6E). Strong iNOS expression was observed in the Control group, whereas BZ treatment markedly reduced the fluorescence intensity. Notably, the BZB group exhibited the lowest iNOS expression among all groups, which was further confirmed by quantitative fluorescence analysis (Figure 6C). These results are consistent with the RT-qPCR findings and provide additional protein-level evidence that BZB effectively suppresses macrophage inflammatory activation and alleviates the pro-inflammatory microenvironment.

The anti-inflammatory effects of BZB may be closely associated with its coordinated regulation of oxidative stress and inflammatory signaling pathways. Excessive intracellular ROS is known to activate pro-inflammatory cascades, particularly the NF-*κ*B signaling pathway, thereby promoting the transcription of inflammatory cytokines such as TNF-*α*, IL-6 and IL-1*β*. In the present study, the significant reduction of ROS levels following BZB treatment may contribute to the suppression of these pro-inflammatory mediators. In addition, both berberine and Zn-containing components have been reported to exhibit intrinsic immunomodulatory activities, including inhibition of NF-*κ*B activation and regulation of macrophage-associated inflammatory responses. Therefore, the observed anti-inflammatory effects of BZB are likely mediated through the combined antioxidative and immunoregulatory activities of the composite system. Meanwhile, the upregulation of IL-10 further suggests that BZB may contribute to the reconstruction of a pro-regenerative inflammatory microenvironment, potentially favoring the transition toward a reparative macrophage phenotype.

Combined with the previously observed ROS scavenging and cell viability results, it can be inferred that BZB effectively reconstructs a pro-regenerative inflammatory microenvironment by simultaneously alleviating oxidative stress and suppressing pro-inflammatory cytokine release. This coordinated regulation of inflammation and oxidative stress provides a more stable biological foundation for epithelial cell migration and angiogenesis under oral ulcer conditions.

### BZB promotes cell migration and angiogenesis

Angiogenesis and epithelial cell migration are two tightly coupled biological processes during oral ulcer healing [52, 53]. Newly formed microvessels supply oxygen and nutrients to the injured tissue, while the directed migration and spreading of epithelial cells determine the efficiency of reepithelialization at the ulcer surface [54, 55]. Therefore, HUVECs and HOK were employed to evaluate the regulatory effects of BZB on angiogenesis- and epithelial repair-related cellular behaviors.

In the HUVECs scratch assay (Figure 7A and B) and Transwell migration assay (Figure 7C and D), the Control group exhibited a relatively slow migration rate, whereas the BC group showed only a modest enhancement. This effect is mainly attributed to the extracellular matrix-like nanofibrous structure of BC, which supports cell adhesion and spreading. In contrast, the BZ group significantly accelerated HUVECs migration. Notably, the BZB group demonstrated the most pronounced pro-migratory effect, with both the wound closure rate and the number of migrated cells significantly higher than those in all other groups, indicating a superior ability of the composite system to promote endothelial cell mobilization.

**Figure 7:**
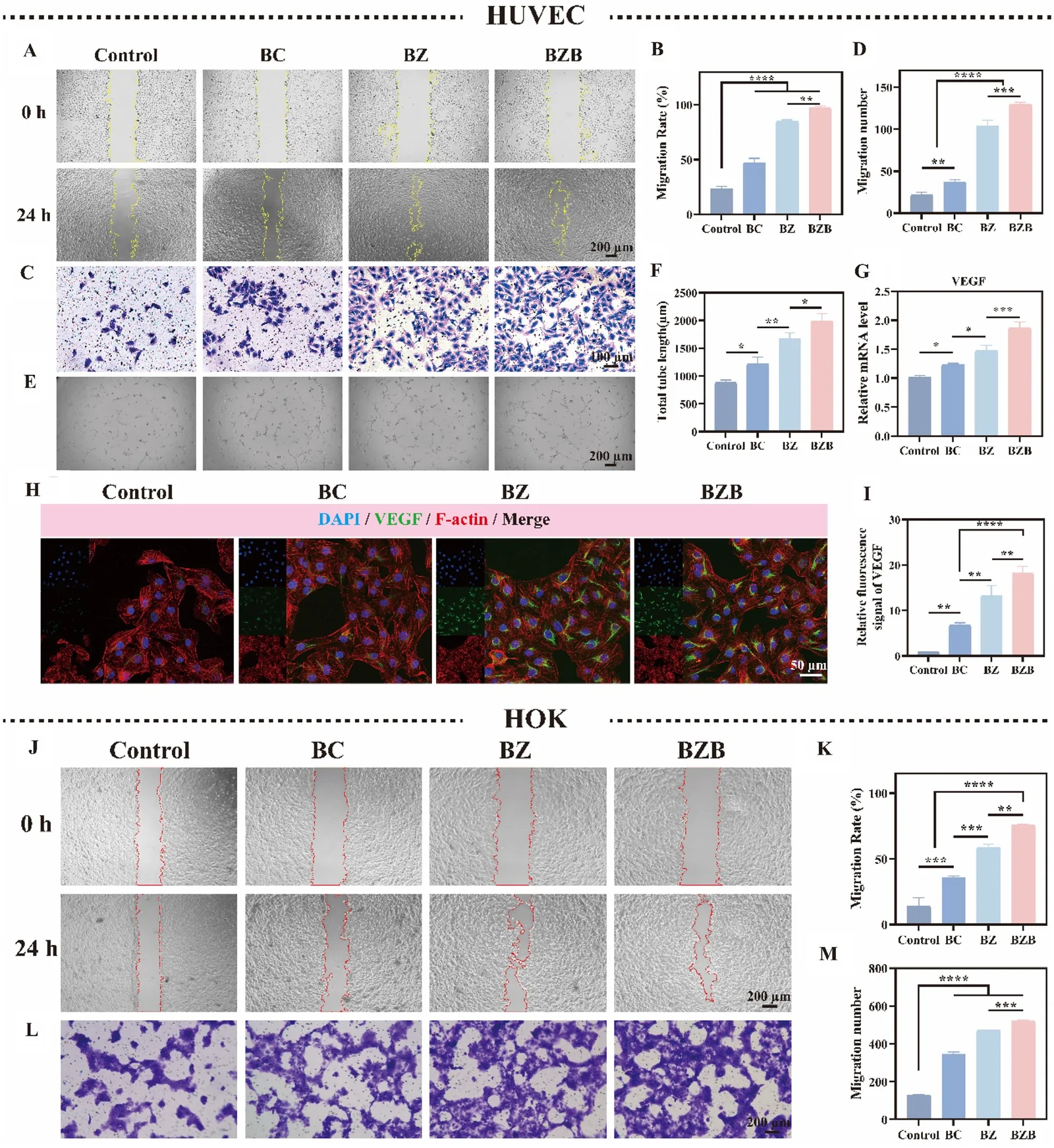
BZB enhances HUVECs and HOK migration and angiogenic activity. (**A**) Scratch assay showing migration of HUVECs. (**B**) Quantification of the scratch assay. (**C**) Transwell migration assay. (**D**) Quantification of migrated cells. (**E**) Tube formation assay demonstrating angiogenic capacity. (**F**) Total tube length with statistical analysis. (**G**) VEGF mRNA expression levels. (**H**) IF staining of VEGF expression. (**I**) Quantitative analysis of IF staining of VEGF expression. (**J**) Scratch assay showing migration of HOK. (**K**) Quantification of the scratch assay. (**L**) Transwell migration assay of HOK. (**M**) Quantification of migrated cells. Data are presented as mean ± SD (*n* = 3 independent experiments). Statistical analysis was performed using one-way ANOVA followed by Tukey’s multiple comparisons test. ns, not significant. *P < 0.05, **P < 0.01, ***P < 0.001 and ****P < 0.0001.

Further tube formation assays (Figure 7E) revealed that the Control group formed only sparse and incomplete capillary-like structures. The BC group showed slight improvement, but the vascular network remained poorly organized with limited branching. In comparison, the BZ group markedly enhanced tubular structure formation. More importantly, the BZB group induced the formation of a denser, more continuous and highly branched vascular-like network. Quantitative analysis of total tube length further confirmed these observations, showing that the BZB group exhibited the greatest degree of network formation among all groups (Figure 7F). These results demonstrate that the stable and sustained delivery of BZ within the BC matrix more effectively activates the angiogenic phenotype of endothelial cells and promotes vascular network assembly.

To further investigate the molecular basis underlying the pro-angiogenic activity of BZB, VEGF expression was evaluated at both the protein and gene levels. IF analysis (Figure 7H and I) demonstrated that VEGF expression was significantly enhanced following BZ treatment and was further increased in the BZB group, which exhibited the highest fluorescence intensity among all groups. Consistent with the protein-level results, RT-qPCR analysis (Figure 7G) revealed a significant upregulation of VEGF mRNA expression in HUVECs after BZB treatment compared with the Control, BC and BZ groups. These findings indicate that BZB not only enhances endothelial angiogenic behavior but also promotes angiogenesis-related gene expression, thereby providing stronger mechanistic evidence for its pro-angiogenic activity. This enhanced effect is likely attributed to the more sustained and stable local release of BZ within the BC matrix, enabling continuous action of Zn^2+^ and berberine on endothelial cells.

A similar trend was observed in HOK scratch and migration assays (Figure 7J–M). The Control group showed slow wound closure, whereas the BC group exhibited a slightly improved migration rate due to its extracellular matrix-like nanofibrous topology, which supports cell adhesion and spreading. The BZ group further accelerated epithelial cell migration. Notably, the BZB group demonstrated the most pronounced promotive effect, achieving the fastest and most complete wound closure within the same time frame. These results indicate that, during epithelial repair associated with oral ulcers healing, BZB not only provides a favorable physical scaffold but also continuously regulates cellular behavior through functional delivery.

Collectively, these findings demonstrate that BZB integrates the pro-angiogenic activity of BZ with the favorable cytocompatibility and interfacial stability of the BC matrix. This synergistic system significantly enhances both migration and angiogenesis in endothelial cells and oral epithelial cells, thereby providing an optimized microenvironment for tissue regeneration under oral ulcer conditions.

### Therapeutic efficacy of BZB in a rat oral ulcer model

To systematically evaluate the *in vivo* therapeutic potential of BZB for oral mucosal repair, a rat oral ulcer model was established using repeated acetic acid stimulation. This model reliably induces epithelial disruption, inflammatory cell infiltration and tissue necrosis, thereby recapitulating the typical pathological features of clinical oral ulcers. Animals were treated with Control, BC, BZB and a commercially available propolis oral membrane.

Macroscopic observations of ulcer areas over time (Supplementary Figure S6) revealed that the Control group maintained clearly defined ulcer boundaries, with persistent necrotic tissue and inflammatory exudates, indicating a slow healing process. The BC group showed moderate ulcer contraction and partial surface repair, reflecting its role as a physical barrier and extracellular matrix-like scaffold that provides baseline support for mucosal regeneration. The propolis-treated group exhibited accelerated ulcer closure compared with the Control group, demonstrating a certain therapeutic benefit. Notably, the BZB group displayed the most pronounced ulcer contraction, with the fastest reduction in wound area and the highest degree of reepithelialization, indicating superior therapeutic efficacy compared with both BC and the commercial propolis membrane (Figure 8A–C).

**Figure 8:**
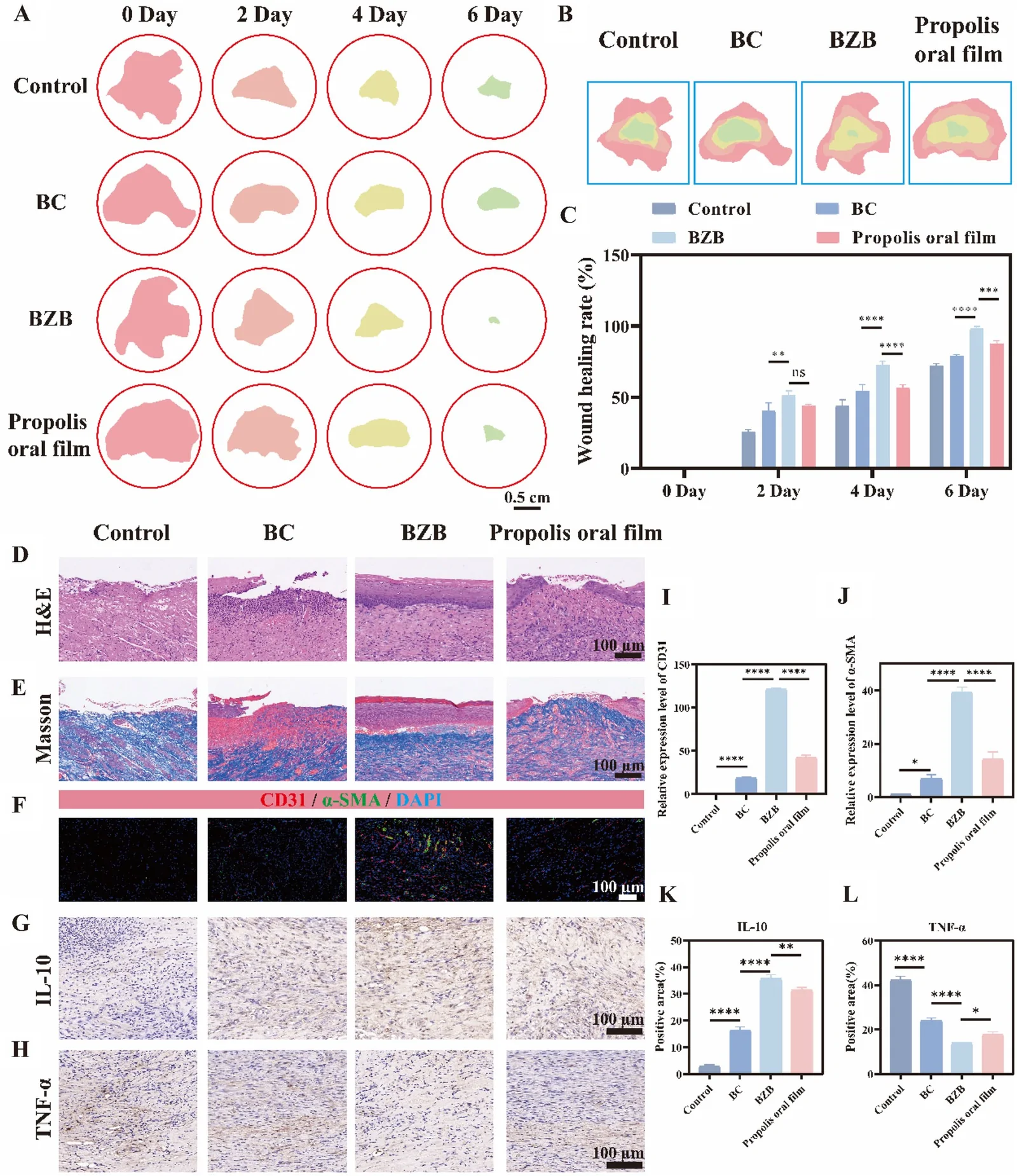
*In vivo* therapeutic efficacy of BZB in a rat oral ulcer model. (**A**) Simulation of wound morphological transformations over a span of 6 days. (**B**) Schematic representation of ulcer healing progression. (**C**) Wound closure rate. (**D**) H&E staining. (**E**) Masson staining. (**F**) CD31 and *α*-SMA staining for angiogenesis and vascular maturation. IHC staining of (**G**) IL-10 and (**H**) TNF-α. (**I, J**) Quantitative analysis of neovascularization. (**K, L**) Quantitative analysis of IHC staining of IL-10 and TNF-*α*. Data are presented as mean ± SD (*n* = 5 independent experiments). Statistical analysis was performed using one-way ANOVA followed by Tukey’s multiple comparisons test. ns, not significant. *P < 0.05, **P < 0.01, ***P < 0.001 and ****P < 0.0001.

H&E staining (Figure 8D) showed that the Control group exhibited severe epithelial loss, accompanied by extensive inflammatory cell infiltration and pronounced edema in the lamina propria. In the BC group, partial epithelial regeneration was observed, although epithelial continuity remained incomplete and inflammation persisted. The propolis group demonstrated improved epithelial repair and reduced inflammation. In contrast, the BZB group exhibited nearly complete epithelial coverage, well-organized mucosal architecture and markedly reduced inflammatory infiltration, indicating effective restoration of tissue structure.

Masson staining further demonstrated (Figure 8E) that the Control group showed disorganized collagen fibers with insufficient deposition. Both the BC and propolis groups exhibited moderate improvement in collagen formation. In comparison, the BZB group presented a denser and well-organized collagen network, suggesting significantly enhanced extracellular matrix reconstruction and tissue remodeling.

CD31 and *α*-SMA staining were performed to evaluate neovascularization and vascular maturation (Figure 8F). In the Control group, only a few scattered CD31 positive microvessels were observed, accompanied by weak *α*-SMA signals, indicating limited angiogenesis. The BC and propolis groups showed an increased number of CD31 positive vessels; however, vascular maturity remained insufficient. In contrast, the BZB group exhibited a markedly higher density of CD31 positive microvessels, along with significantly enhanced *α*-SMA positive smooth muscle coverage. These results indicate that BZB not only promotes neovascular formation but also facilitates vascular maturation and stabilization, which are essential for long-term mucosal repair (Figure 8I and J).

IHC analysis further revealed (Figure 8G and H) that the Control group displayed strong TNF-*α* expression and relatively low IL-10 levels, representing a persistent pro-inflammatory state. In the BC and propolis groups, TNF-*α* expression was moderately reduced, while IL-10 levels were slightly increased. Notably, the BZB group showed a significant suppression of TNF-*α* expression along with a pronounced upregulation of IL-10. Quantitative analysis of the IHC staining further confirmed these observations, showing significantly lower TNF-*α* levels and higher IL-10 levels in the BZB group compared with the other treatment groups (Figure 8K and L). These results indicate that this patch effectively modulates the inflammatory microenvironment and promotes a shift from a pro-inflammatory state toward a pro-regenerative immune phenotype.

In addition, the *in vivo* biosafety of BZB was further evaluated by histological examination of major organs, including the heart, liver, spleen, lung and kidney. As shown in Supplementary Figure S7, H&E staining revealed normal tissue architecture without observable pathological abnormalities in all groups. The morphology and organization of cells in these organs remained comparable to those of healthy tissues, indicating that neither BC nor BZB induced detectable systemic toxicity. These results collectively demonstrate the favorable *in vivo* biocompatibility and biosafety of BZB, supporting its potential for oral mucosal repair applications.

Regarding biodegradation and long-term biosafety, BZB is designed as a temporary local therapeutic patch for short-duration oral ulcer treatment rather than a permanent implantable biomaterial. Since oral ulcer healing is typically completed within ∼1 week, maintaining temporary structural persistence during the therapeutic period is considered beneficial for sustained local drug retention and barrier protection in the highly dynamic oral environment. In addition, the therapeutically active BZ component gradually releases berberine and Zn^2+^ under inflammatory conditions, while berberine and Zn^2+^ may subsequently participate in physiological metabolism. Nevertheless, more comprehensive long-term degradation and chronic biosafety evaluations remain necessary in future translational studies. The temporary structural persistence and sustained therapeutic regulation of BZB are highly compatible with the short-term healing characteristics of oral ulcers, highlighting its translational potential as a localized therapeutic patch for oral mucosal repair.

Collectively, these *in vivo* results demonstrate that BZB accelerates ulcer healing through a synergistic mechanism. The Janus BC matrix provides stable adhesion and an extracellular matrix-like scaffold to maintain local microenvironmental stability. Meanwhile, BZ enables sustained release of berberine and Zn^2+^, contributing to anti-inflammatory effects and activation of angiogenesis. The combined modulation of the local microenvironment further promotes epithelial regeneration and extracellular matrix remodeling. Compared with BC or the commercial propolis membrane, BZB exhibits superior therapeutic performance in terms of inflammation suppression, angiogenesis promotion and tissue reconstruction, highlighting its strong potential for clinical translation as a precision therapeutic patch for oral ulcers.

Despite the favorable therapeutic efficacy observed in this study, several limitations should still be acknowledged. The acetic acid-induced oral ulcer model used herein primarily represents an acute chemically induced mucosal injury model and therefore cannot fully recapitulate the complex immunopathological mechanisms, recurrent characteristics and multifactorial etiology of clinically common recurrent aphthous ulcers and other chronic oral ulcerative disorders. In addition, the current evaluation mainly focused on short-term therapeutic performance and biosafety, while the long-term degradation behavior, chronic oral exposure compatibility and metabolic fate have not yet been comprehensively investigated. Therefore, future studies should further employ more clinically relevant recurrent or immune-associated ulcer models and conduct long-term biosafety and degradation assessments to better evaluate the translational potential of the BZB system for oral mucosal therapy.

## Conclusion

In this study, a Janus-structured BC composite patch, BZB, was developed to modulate the complex pathological microenvironment of oral ulcers. By integrating BZ into the Janus BC matrix, the system sustained and localized drug release. BZB effectively inhibited bacterial colonization, reduced intracellular ROS levels and preserved cell viability under oxidative stress conditions, while simultaneously promoting epithelial and endothelial cell migration as well as angiogenesis. In a rat oral ulcer model, BZB significantly accelerated ulcer closure, improved collagen remodeling, facilitated vascular network reconstruction and modulated the inflammatory microenvironment toward a pro-healing state. These findings demonstrate that BZB can simultaneously target multiple pathological factors involved in oral ulcer progression, thereby providing a more comprehensive therapeutic strategy than conventional topical treatments.

Given its favorable bioactivity, prolonged local retention and multifunctional microenvironment-regulating capability, BZB shows considerable potential as a next-generation bioactive patch for the treatment of oral ulcers and other oral mucosal lesions characterized by infection, oxidative stress and persistent inflammation. It should be noted that the acetic acid-induced oral ulcer model employed in this study primarily represents an acute mucosal injury model and does not fully reproduce the immune dysregulation and recurrent nature of clinically common recurrent aphthous stomatitis. Nevertheless, excessive inflammation, oxidative stress, bacterial colonization and impaired tissue regeneration are shared pathological features between acute and recurrent ulcers. Therefore, the present model remains suitable for evaluating the local therapeutic efficacy of biomaterial-based interventions. Future investigations should focus on recurrent and chronic ulcer models, long-term biosafety and degradation behavior, large-animal validation and manufacturing optimization to further facilitate clinical translation of this promising therapeutic platform.

## Supplementary Material

rbag160_Supplementary_Data

## Data Availability

Data will be made available on request.
