# Supplementary material for: A Janus bacterial cellulose patch with antibacterial, antioxidant, anti-inflammatory and angiogenic activities for enhanced oral ulcer healing
Source: Regen Biomater. 2026 Jul 25;13:rbag160. doi: 10.1093/rb/rbag160 (PMC13653682; doi:10.1093/rb/rbag160)
Supplement: Supplementary file 1 [file rbag160_supplementary_data.docx]

**Supporting information**

**A Janus Bacterial Cellulose Patch with Antibacterial, Antioxidant, Anti-Inflammatory and Angiogenic Activities for Enhanced Oral Ulcer Healing**

Guning Wang ^a,1^, Jingyu Yan^a,1^, Chenying Cui^a,1^, Lihong Zhou^a^, Xiuping Wu^a,^*, Bing Li^a,^*

Figure S1. SEM images of ZIF.

Figure S2. SEM images of BZB after incubation in artificial saliva. The porous drug-loading layer (A) and dense adhesive layer (B) showed preserved Janus architecture and nanofibrous morphology.

Figure S3. RWR of BC and BZB after treatment under artificial saliva conditions.

Figure S4. Standard calibration curve of berberine showing the linear relationship between absorbance and concentration.

Figure S5. Adhesion strength of BC and BZB patches after artificial saliva soaking, stirring, and sonication treatments.

Figure S6. Representative images of ulcer healing progression.

Figure S7. Histological analysis of major organs after different treatments.

**Table**

**Table S1.** Primers used in the RT-qPCR analysis.

| Genes | Forward primer | Reverse primer |
| --- | --- | --- |
| TNF-*α* | CTTGTTGCCTCCTCTTTTGCTTA | CTTTATTTCTCTCAATGACCCGTAG |
| IL-6 | ATA ACC ACC CCT GAC CCA AC | CCC ATG CTA CAT TTG CCG AA |
| IL-1*β* | TGTGTTTTCCTCCTTGCCTCTGAT | TGCTGCCTAATGTCCCCTTGAAT |
| IL-10 | GGAAGACAATAACTGCACCCACT | CAACCCAAGTAACCCTTAAAGTCC |
| VEGF | GTCCTCTCCTTACCCCACCTCCT | CTCACACACACAGCCAAGTCTCCT |
| GAPDH | AGAAGGTGGTGAAGCAGGCATC | CGAAGGTGGAAGAGTGGGAGTTG |
